# Functional determinants of lysophospholipid- and voltage-dependent regulation of TRPC5 channel

**DOI:** 10.1007/s00018-024-05417-7

**Published:** 2024-08-29

**Authors:** Alexandra Ptakova, Lucie Zimova, Ivan Barvik, Robin S. Bon, Viktorie Vlachova

**Affiliations:** 1 Department of Cellular Neurophysiology, Institute of Physiology, Czech Academy of Sciences, Videnska 1083, 142 20 Prague 4, Czech Republic; 2 Department of Physiology, Faculty of Science, Charles University, Prague, Czech Republic; 3 Division of Biomolecular Physics, Institute of Physics, Faculty of Mathematics and Physics, Charles University, Prague, Czech Republic; 4 Leeds Institute of Cardiovascular and Metabolic Medicine (LICAMM) and Astbury Centre for Structural Molecular Biology, University of Leeds, Leeds, LS2 9JT UK

**Keywords:** TRP channels, Voltage-dependent gating, Lysophosphatidylcholine, Pain, TRPC channels

## Abstract

Lysophosphatidylcholine (LPC) is a bioactive lipid present at high concentrations in inflamed and injured tissues where it contributes to the initiation and maintenance of pain. One of its important molecular effectors is the transient receptor potential canonical 5 (TRPC5), but the explicit mechanism of the activation is unknown. Using electrophysiology, mutagenesis and molecular dynamics simulations, we show that LPC-induced activation of TRPC5 is modulated by xanthine ligands and depolarizing voltage, and involves conserved residues within the lateral fenestration of the pore domain. Replacement of W577 with alanine (W577A) rendered the channel insensitive to strong depolarizing voltage, but LPC still activated this mutant at highly depolarizing potentials. Substitution of G606 located directly opposite position 577 with tryptophan rescued the sensitivity of W577A to depolarization. Molecular simulations showed that depolarization widens the lower gate of the channel and this conformational change is prevented by the W577A mutation or removal of resident lipids. We propose a gating scheme in which depolarizing voltage and lipid-pore helix interactions act together to promote TRPC5 channel opening.

**Supplementary Information:**

The online version contains supplementary material available at 10.1007/s00018-024-05417-7.

## Introduction

The current development of advanced lipidomic techniques provides increasing evidence that the pro-inflammatory lipid lysophosphatidylcholine (LPC) is a potential direct endogenous mediator contributing to the generation and maintenance of pain [1]. This signaling lipid is present at high concentrations in painful inflammatory exudates and can activate and/or sensitize various pain-related ion channels, including acid-sensing ion channel ASIC3 [2], two-pore domain potassium channels K_2P_2.1 and K_2P_4.1 [3], and several members of the TRP (transient receptor potential) channel family [4–9]. Because targeting LPC and its metabolism is emerging as a promising potential therapeutic approach for the treatment of inflammation and pain-related diseases, determining the precise molecular mechanism of activation of these channels becomes an urgent task.

One of the important LPC-activated channels whose role in pain has only recently been demonstrated is the transient receptor potential canonical 5 (TRPC5) [10, 11]. This polymodal calcium-permeable cation channel is expressed in various tissues including the central and peripheral nervous system. The channel can be activated by (-)-englerin A (EA), a selective, sesquiterpene-based TRPC4/5 agonist [12, 13], which is hypothesized to bind to a lipid-recognition site within the pore domain [14, 15]. This region, also referred to as the L2 lipid site, forms a lateral hydrophobic fenestration between the fifth transmembrane segment S5 and the pore helix from one subunit and the sixth segment S6 of the adjacent subunit [14, 16, 17]. It is capable of accommodating diacylglycerol (DAG), a major physiological activator that controls the gating of most TRPC channel family members [17–19]. The “LFW” motif inside the pore helix and the directly opposite glycine residue in S6, conserved among TRPCs, are involved in lipid recognition [18, 20–22]. Within the LFW motif, the aromatic residues F576 and W577 form a π-π interaction that stabilizes the pore region and their individual alanine substitutions reduce the potency of EA by two to three orders of magnitude [14], whereas mutations of the entire motif render the TRPC5 channel inactive [16, 21, 23]. The glycine located directly opposite to W577 has previously been shown to be an integral part of the conserved lipid-recognition mechanism in the related channel TRPC3 [18]. Substitution of this glycine (G652 in TRPC3 corresponding to G606 in TRPC5) with the larger leucine resulted in a loss of function and modified the channel’s ability to discriminate among different lipid mediators. Moreover, the G652L mutant retained sensitivity of TRPC3 to the small molecule activator GSK1702934A, which argued against a more generalized role for this glycine in the gating movements downstream of lipid recognition [18]. The potent and selective xanthine-based inhibitor of TRPC1/4/5 channels Pico145 [24] binds also to the conserved L2 lipid site, where it displaces a resident (phospho)lipid that interacts with the pore helix near the extracellular side [14]. Near the entrance to the extracellular pore of the channel is a binding site for cations (E543 and E595), through which protons and lanthanides (La^3+^ and Gd^3+^) markedly enhance TRPC5 activity [25, 26]. In addition, TRPC5 behaves as a voltage-gated channel, since it can be activated, in the absence of any agonist, by depolarizing voltages (> + 60 mV) [27], but the mechanism is currently unknown. In native tissues, TRPC5 regulation is complex and involves a variety of different stimuli, such as changes in the redox state, pH or intracellular Ca^2+^ concentration, G-proteins and their downstream signaling pathways, cold, and osmo-mechanical stimuli [28–31]. Although TRPC5 is dispensable for normal mechanical sensation, it is a major contributor to tactile and spontaneous pain, which are intractable symptoms of inflammatory and neuropathic injury [10]. In mouse models, TRPC5 selectively contributes to persistent mechanical hypersensitivity associated with skin incision, chemotherapy-induced peripheral neuropathy, sickle cell disease, complete Freund’s adjuvant injection, and migraine. Importantly, all of these conditions are associated with elevated concentrations of LPC [10], which is a powerful activator of TRPC5 [7]. However, the explicit molecular mechanism by which the activation occurs is largely unknown. The original study by Flemming and colleagues, which first discovered TRPC5 activation by LPC, demonstrated that LPC activates TRPC5 in a manner that depends on its carbon chain length and supported a direct interaction mechanism while carefully ruling out a number of possibilities for an indirect effect [7, 32].

Since the LPC molecule is a conical lipid, its asymmetric incorporation into the plasma membrane may induce mechanical deformation [33, 34], thereby activating the channels in their surrounding membrane microenvironment without interacting through a specific binding site. On the other hand, a direct interaction of LPC with some channels has been proposed based on mutagenesis, using different situations in which channel activity was measured, and comparing the effects of LPC with trinitrophenol, another amphipatic molecule known to induce membrane deformation [2, 6, 32]. The increasing number of high-resolution structures of TRP ion channels in different conformational states obtained in the last few years indicates that different physiological lipids occur at functionally key sites and likely represent endogenous ligands and/or compete with them in channel regulation. Some of these are even required at a specific point in the gating cycle of the channel [35]. However, due to the intrinsic malleability of the interaction sites, the identity and precise location of the lipids remain frequently ambiguous and do not indicate whether the site of potential interaction is functionally significant [36, 37]. Therefore, refined functional examination in combination with structure- and simulation-guided mutagenesis remains an essential way to determine the mechanism of LPC activation. Here, we set out to better understand the molecular mechanism through which LPC activates human TRPC5 and explore the possibility that it directly interacts with the channel to stabilize its open state.

## Results

### LPC modulates voltage-dependent gating of TRPC5

For this study, we chose LPC 18:1 because: first, LPC 18:1, together with LPC 16:0 and LPC 18:0, are the most abundant forms found to activate TRP channels in various painful and inflammatory conditions [5, 9, 10, 38]; second, TRPC5 activation requires a chain length greater than 12 [7]; and third, LPC 18:1 has better aqueous solubility than LPC 18:0 and is a more efficacious TRPC5 activator than LPC 16:0 [7].

**Fig. 1:**
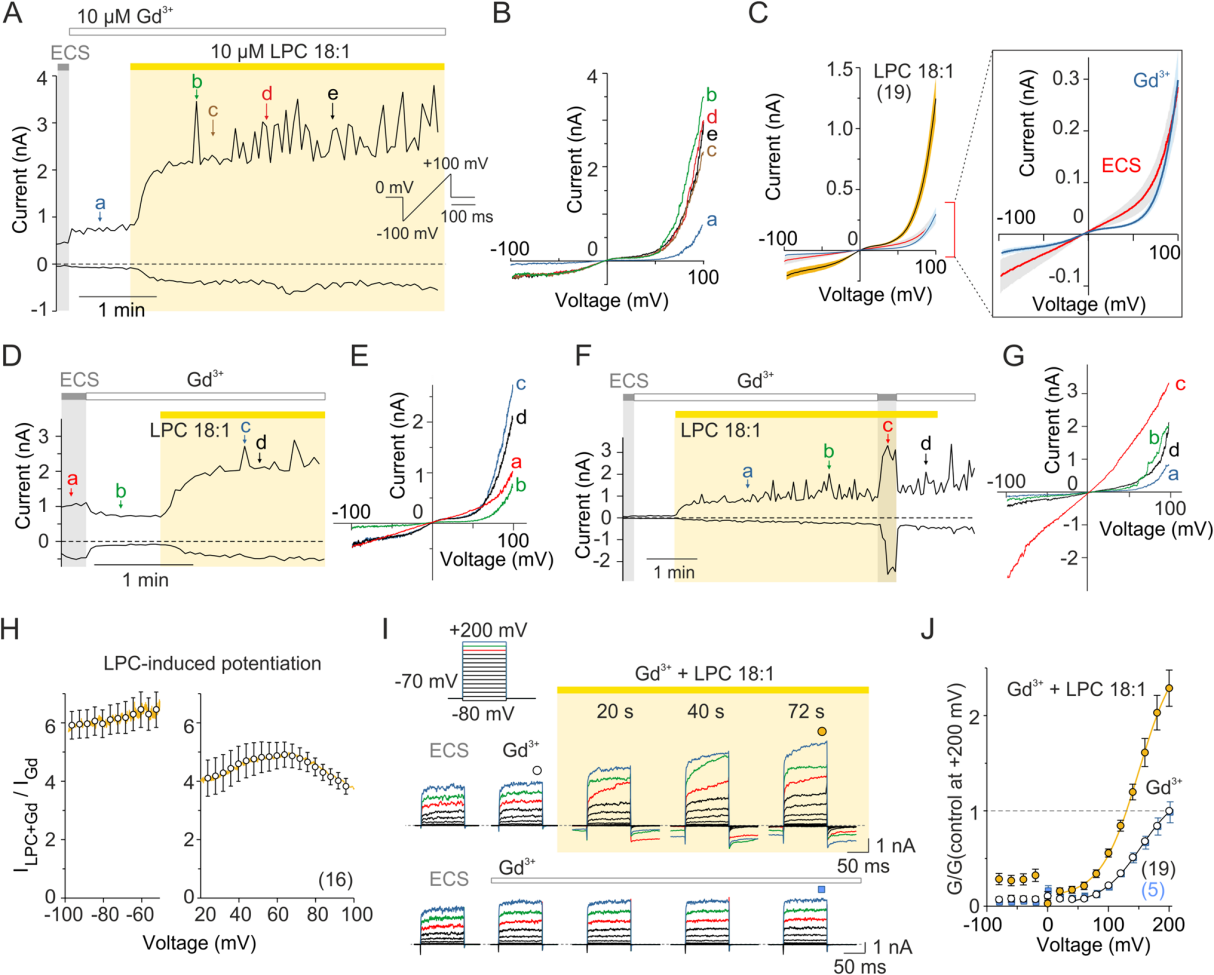
Lysophosphatidylcholine 18:1 activates human TRPC5 in HEK293T cells and modulates voltage-dependent gating. **A** Representative whole-cell currents recorded from a TRPC5-expressing HEK293T cell in extracellular control solution (ECS) not containing and then containing Gd^3+^ (10 µM), followed by the addition of 10 µM LPC (LPC 18:1). A ramp pulse from − 100 mV to + 100 mV from a holding potential of 0 mV was periodically applied every 3 s for 500 ms (inset). Amplitudes were measured at − 100 mV and + 100 mV and plotted as a function of time. **B** The current–voltage relations at the time points indicated by the letters in panel A. **C** Mean current–voltage relations averaged over ~ 1 min of steady-state LPC application (mean as solid lines, ± SEM as lighter-colored envelopes; *n* = 19) plotted for the currents recorded as shown in A. Right, zoomed view of the effect of 10 µM Gd^3+^ (blue line; ± SEM as light-blue envelopes; *n* = 19) on endogenous voltage-induced currents measured in extracellular control solution (ECS; red line; ± SEM as light-gray envelopes). The amplitudes of currents measured in the absence of Gd^3+^ at − 100 mV (− 87 ± 27 pA) and + 100 mV (283 ± 62 pA) were not significantly different from the current amplitudes measured in the presence of Gd^3+^ (− 49 ± 15 pA and 298 ± 58 pA; *P* = 0.863 and *P* = 0.238; *n* = 19; paired *t* test). **D**,** F** Representative whole-cell currents recorded from a TRPC5-expressing HEK293T cell in ECS not containing and then containing 10 µM Gd^3+^, followed by the addition of 10 µM LPC. A ramp pulse protocol was used as in panel A. Amplitudes were measured at − 100 mV and + 100 mV and plotted as a function of time. **E**, **G** The current–voltage relations at the time points indicated by the letters in panels D and F. **H** Average increase in voltage-dependent currents induced by LPC using a voltage protocol as in A. At a membrane potential of − 80 mV, the currents were potentiated 6.0 ± 0.5-fold by LPC, but only 4.5 ± 0.3-fold at + 80 mV (*n* = 16). **I** Representative current traces in response to a 100-ms voltage step family from − 80 to + 200 mV (20 mV step; inset) recorded from TRPC5-expressing cells. The currents were recorded in control solution ~ 1 min after whole-cell formation, after 30–40 s of exposure to 10 μM Gd^3+^, and after 1–2 min of exposure to LPC (upper traces) or 10 µM Gd^3+^, as a control (lower traces). Steady-state currents were measured at the end of the pulses as indicated by colored symbols atop the records. Note the fluctuations at higher (≥ 160 mV) potentials indicated by colored traces in LPC treated cells. **J** The average conductance-voltage plots normalized to the maximum response to + 200 mV obtained in extracellular control solution containing Gd^3+^. The data were fitted by Boltzmann equation over the interval + 40 mV to + 200 mV; solid lines). Number of biological replicates is indicated in parentheses (*n* = 19 for cells exposed to LPC; empty circles, black errors bars are smaller than the symbols for most of the data points, and *n* = 5 for cells treated with Gd^3+^ without LPC; blue squares and error bars). Data are mean ± SEM

We used a clinically relevant concentration of 10 µM LPC 18:1 (LPC) and measured membrane currents from human embryonic kidney (HEK) 293T cells transiently transfected with human TRPC5 by whole-cell electrophysiology. Voltage ramps of 500 ms duration from − 100 mV to + 100 mV were delivered periodically every 3 s from a holding potential of 0 mV, or 100-ms depolarizing pulses from − 80 mV to + 200 mV were applied from a holding potential of − 70 mV (Fig. 1). As previously used and reasoned in [7], the experiments were carried out in the presence of Gd^3+^ (10 µM) to eliminate any contribution of LPC-activated endogenous currents present in 293T cells (Fig. 1C–G and Supplementary Fig. S1A-E). At the concentration used, Gd^3+^ blocked background currents and its weak sensitizing effect on TRPC5 was seen in only those cells, in which significant background was not present (see Fig. 1A–C and compare contrasting representative traces in Fig. 1D–G and Supplementary Fig. S1A-E). Stimulation of TRPC5-expressing HEK293T cells with LPC produced large currents with the typical double rectifying current–voltage relationship (Fig. 1B, C), but induced only very small effects in non-transfected cells (Supplementary Fig. S1F, G). The half-maximal concentration for LPC-induced TRPC5 activation was 0.53 µM at − 100 mV (Hill coefficient, *h* = 0.93), and 0.37 µM at + 100 mV (*h* = 0.86; *n* = 9) (Supplementary Fig. S1J–L). LPC at a concentration of 10 µM potentiated the TRPC5-mediated currents in a voltage-dependent manner: the half-maximal voltage (*V*_50_) of steady-state activation decreased from 153.8 ± 3.6 mV to 139.0 ± 3.9 mV (*n* = 18) and the apparent number of gating charges (*z*) increased from 0.76 ± 0.03 e_0_ to 0.95 ± 0.1 e_0_ (Supplementary Fig. S2A–C). At positive membrane voltages, the LPC-induced potentiation peaked at + 60 mV, whereas at negative membrane potentials the potentiation was nearly voltage independent (Fig. 1H). To further explore the voltage dependence of the LPC-induced effects, we next compared the steady-state activation curves for TRPC5 currents elicited by a voltage step protocol in the presence of 1, 3 and 10 µM LPC (Supplementary Fig. S2D–F). It was evident that the average values of *V*_50_ decreased with increasing concentration of LPC from 1 µM to 3 µM, indicating that LPC acts by shifting the voltage dependence of the channel towards positive membrane potentials. It was striking that in the presence of 10 µM LPC, increases in outward currents were frequently observed at strongly (> + 80 mV) depolarizing potentials, which was manifested as fluctuations when peak amplitudes were plotted over time (see Fig. 1A, B and I). This was unlikely to be due to a transient deterioration in recording quality or membrane disruption/lysis by the lipid, as there was a rapid recovery of current after the voltage returned to lower values. This phenomenon was present during both linear voltage ramp stimulation (Fig. 1A) and the voltage step protocol application (Fig. 1I). This effect of LPC has not been previously described and may be due to a non-specific action on the channel. However, it may also be a manifestation of a specific functional interaction of LPC with a putative voltage-sensitive region that cooperatively regulates channel activity during depolarization. Alternatively, at high positive potentials, the Gd^3+^ cations can detach from the extracellular cation binding site of TRPC5 (i.e. E543 and E595), which subsequently reduces the current potentiation. We further explored these alternatives.

### Pico145 reduces LPC-induced responses; (−)-englerin A functionally interacts with LPC

**Fig. 2:**
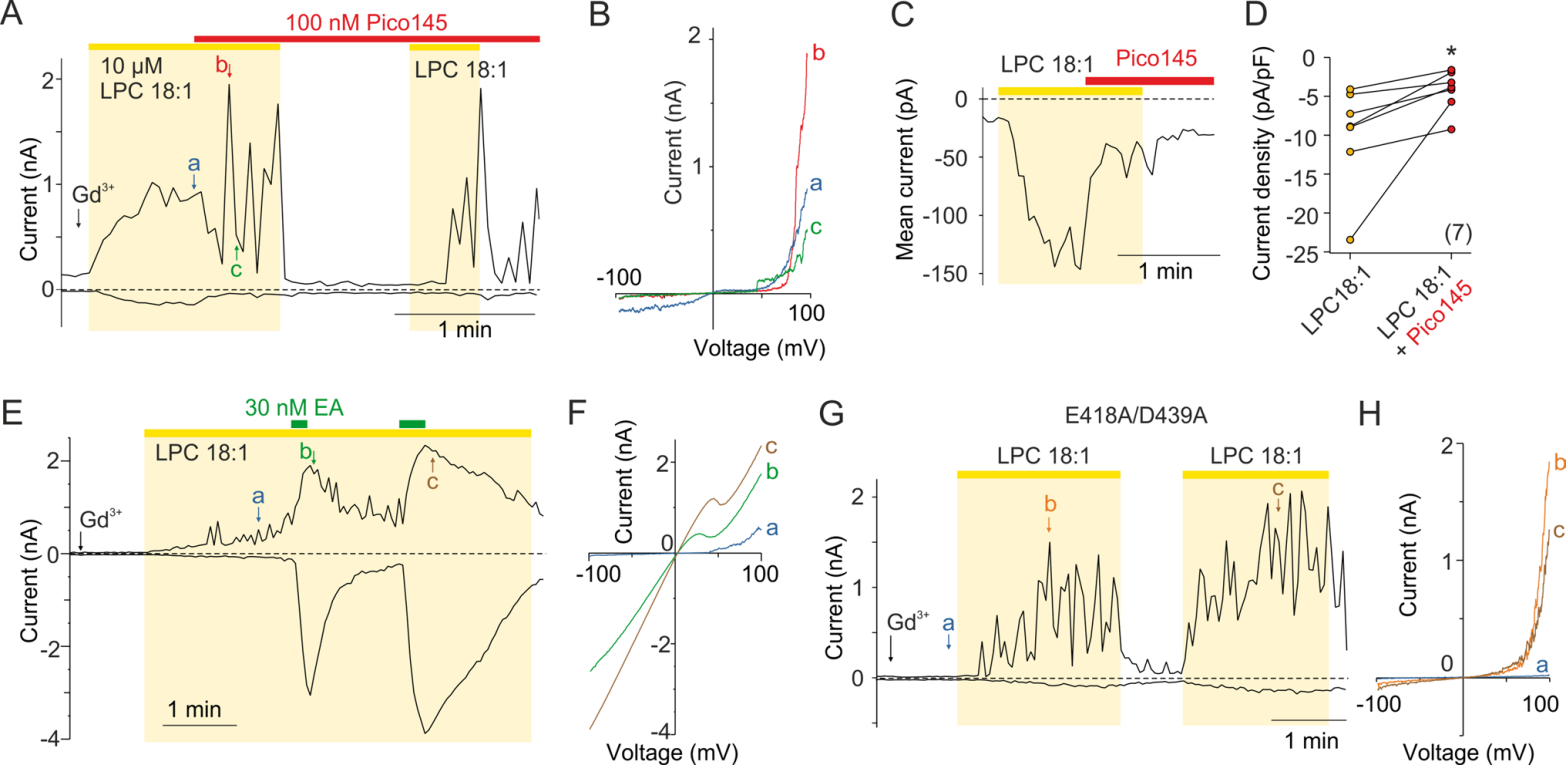
LPC-induced responses are blocked by Pico145 and modulated by (-)-englerin A. **A** Representative recording from a TRPC5-expressing HEK293T cell exposed to LPC (LPC 18:1; 10 µM) in extracellular control solution containing Gd^3+^ (10 µM; the beginning of exposure is indicated by vertical arrow), followed by 100 nM Pico145. A ramp pulse protocol as shown in Fig. 1A was applied. Amplitudes were measured at − 100 mV and + 100 mV and plotted as a function of time. **B** The current–voltage relations at the time points indicated by the colored letters in panel A. For c and b, note the fluctuations above + 40 mV. **C** Representative time course of TRPC5-mediated LPC-evoked current inhibition induced by 100 nM Pico145 measured at − 90 mV. **D** Statistics of the Pico145-induced inhibition of LPC responses in seven TRPC5-expressing cells measured at − 90 mV as shown in C. Student's paired *t* test (*P < 0.05). **E** (−)-englerin A (EA; 30 nM) induces robust currents in the presence of LPC and suppresses current fluctuations at the depolarizing potential during long-term application. **F** The current–voltage relations at the time points indicated by the colored letters in panel E. **G** Representative time course of LPC-induced currents in the double mutant of TRPC5, in which the negatively charged residues involved in Ca^2+^ binding localized in the intracellular cavity of the sensor domain were neutralized (E418A /D439A). Similar observations were made for six other cells. **H** The current–voltage relations at the time points indicated by the colored letters in panel G

The responses of TRPC5-expressing cells to LPC at negative membrane potentials were effectively inhibited by the selective xanthine-based inhibitor Pico145 (Fig. 2A–D). At a concentration of 100 nM, this compound reduced the currents to 47.2 ± 7.9% at negative holding potentials (− 90 mV; *n* = 7); however, above + 80 mV, the transient cooperative increases in outward currents were more pronounced and often reached higher amplitudes than for LPC alone (Fig. 2A and B). This effect is consistent with the voltage-dependent action of Pico145 in the presence of Gd^3+^ previously described for TRPC4 and TRPC5 [15, 24]. Subsequent washout of LPC in the presence of Pico145 reduced the activity to near basal levels and LPC was then still able to reactivate the channels. These data provide a strong argument supporting a specific interaction of LPC with TRPC5 and suggest an involvement of the conserved lipid- and xanthine-binding site that is essential to channel gating [14].

Next, we applied LPC together with (-)-englerin A (EA). Brief application of 30 nM EA (~ 10 s) activated large currents in the presence of LPC (5.5 ± 1.1-fold increase at + 90 mV; *n* = 13), but amplitude fluctuations still occurred at positive potentials. In contrast, these were not present if EA was applied for a sufficiently long period of time (30 s) (Fig. 2E and F). The currents produced by EA together with LPC were effectively inhibited by 100 nM Pico145 (Supplementary Fig. S3A-C). Comparing the effects of LPC on partially and fully activated channels at positive potentials (Fig. 2E) indicates that LPC is unable to fully activate TRPC5 and its effect on EA-induced activity is not additive, providing further evidence that LPC and EA may share a common pathway. An alternative explanation may be that a channel whose gating-related activation energy has been reduced by an agonist such as EA or Ca^2+^ passing through the activated channel is no longer sensitive to the physical changes induced by LPC in its surroundings. Therefore, we tried to test the effect of LPC as far as possible without other contributing stimuli. Because TRPC5 is sensitive to sub-micromolar intracellular Ca^2+^ concentrations (100 nM free Ca^2+^ was routinely used in the intracellular solution), we neutralized the two negatively charged residues involved in Ca^2+^ binding, E418 and D439, localized in the intracellular cavity of the sensor domain [16, 17]. The aim was to weaken the Ca^2+^ binding within the channel cavity, thereby attenuating its contribution to LPC responses. LPC still produced large currents mediated by the E418A/D439A double-mutant channels, with responses that were not significantly different from wild-type channels, were rapidly reversible and showed marked amplitude fluctuations at strongly positive potentials (Fig. 2G, H and Supplementary Fig. S7K). Collectively, the results support a very specific, relatively direct and reversible effect of LPC on the channel and suggest that LPC potentiates the voltage-dependent mode of activation of TRPC5.

To explore whether LPC can modulate TRPC5 activity in sensory neuron-like cells, we expressed human TRPC5 in the neuron-derived F11 cell line and measured currents in response to 10 µM LPC 18:1 and 30 nM EA in the absence or presence of 10 µM Gd^3+^ (Supplementary Fig. S3D-E). In non-transfected F11 cells, the application of EA did not induce any response, whereas LPC produced significant currents at positive and negative membrane potentials with near to linear current–voltage characteristics. In F11 cells transfected with TRPC5, LPC elicited responses with linear or slightly double-rectifying current–voltage characteristics, indicating that endogenous targets other than TRPC5 homomers are predominantly involved. The most remarkable observation was that EA-induced currents exhibited dramatically slowed activation and accelerated deactivation kinetics compared to HEK293T cells, indicating different functional properties of TRPC5 in F11 cells.

### LPC activation involves the conserved *glycine* within the lipid-recognition window

**Fig. 3:**
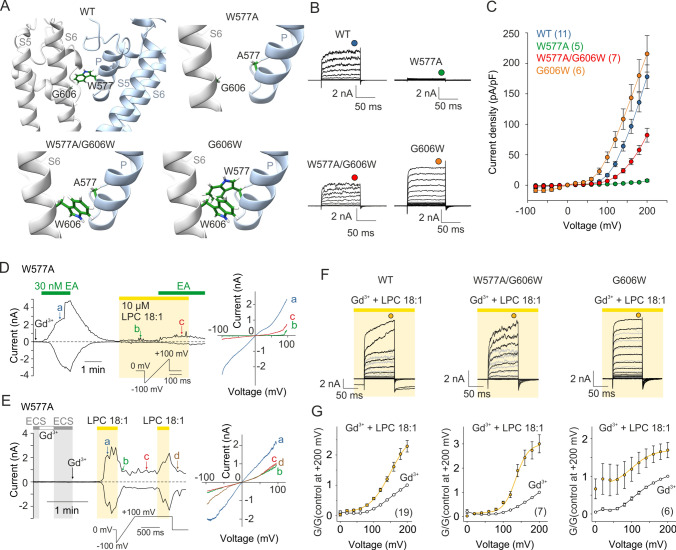
Activation of TRPC5 by LPC 18:1 involves the conserved glycine within the lipid-recognition window. **A** Detailed view of the lipid-recognition window (also termed L2 lipid-binding site) of human TRPC5 (PDB ID: 7E4T) with the residues mutated in this study indicated: W577 in the “LFW” motif inside the pore helix (P) and the directly opposite residue G606 in S6 are shown in stick representation. **B** Representative current traces in response to a voltage step protocol (from − 80 to + 200 mV; as shown in Fig. 1I) recorded from cells expressing wild-type (WT) or mutant TRPC5-channels as indicated above. The currents were recorded in the presence of 10 μM Gd^3+^. **C** The average current density–voltage plots for the indicated TRPC5 constructs. Steady-state currents were measured at the end of the pulses as indicated by colored symbols atop the records shown in B. The lines connecting average data points have no theoretical meaning. Number of biological replicates for each condition is indicated in parentheses. Data are mean ± SEM. **D**,** E** Representative current responses measured in the presence of (−)-englerin A (EA; 30 nM) and/or in the presence of LPC 18:1 (10 µM) in the W577A mutant channels. Ramp pulse protocols are indicated. Amplitudes measured at − 100 mV and + 100 mV were plotted as a function of time. Right, the current–voltage relations at the time points indicated by the colored letters in the left panels. **F** Representative current traces in response to a voltage step protocol from − 80 to + 200 mV (20 mV step) as shown in Fig. 1I, recorded from indicated TRPC5 constructs. The currents were recorded in extracellular solution containing 10 μM Gd^3+^  ~ 1 min after whole-cell formation (light gray traces), and after 1–2 min of exposure to LPC (black traces). **G** The average conductance-voltage plots, normalized to the maximum response at + 200 mV obtained in extracellular solution containing 10 µM Gd^3+^. Steady-state currents were measured at the end of the pulses as indicated by colored symbols atop the records shown in F. The black lines connecting average data points obtained in control solution containing Gd^3+^ (empty circles) have no theoretical meaning; the average data obtained in the presence of LPC 18:1 were fitted by Boltzmann equation (over the interval + 40 to + 200 mV; ochre lines). Number of biological replicates for each condition is indicated in parentheses. Data are mean ± SEM

We further investigated the possibility that LPC acts through the L2 lipid-recognition site [17–19]. Specifically, we focused on the highly conserved “LFW” motif inside the pore helix and the directly opposite glycine residue 606 in S6 (Fig. 3A). Our molecular docking based on the structure of human TRPC5 (7E4T; from which the ligand YZY was extracted) well supported the L2 as a possible interaction site for LPC 18:1 (Supplementary Fig. S4). To experimentally address the role of the L2 lipid-recognition site in the ability of TRPC5 to respond to LPC, we replaced W577 with alanine and measured voltage-dependent responses (Fig. 3). W577A channels were completely insensitive to strong depolarizing voltages (up to + 200 mV) in extracellular solution containing Gd^3+^ (Fig. 3B and C). This mutant responded to EA albeit with a slow kinetics (compare Fig. 3D with Supplementary Fig. S1H and I) and it exhibited only very small responses to LPC. However, when a voltage ramp protocol with a prolonged depolarization phase (+ 100 mV for 1 s) was applied, significant responses to LPC were obtained (Fig. 3E) that reached similar amplitudes to wild-type channels (Supplementary Fig. S7K). The W577A-mediated LPC-induced currents exhibited only weakly double rectifying current–voltage relationship, which we consider to be due to the change in the voltage-dependent blocking action of Mg^2+^ [39] caused by the mutation and/or prolonged depolarization used in this specific experiment. We reasoned that if the W577A mutation destabilizes the pore, the interaction of LPC with its immediate surroundings might in turn have a stabilizing effect and allow channel gating. We therefore investigated this region in more detail.

We hypothesized that replacing the critical glycine 606 in S6 with tryptophan might increase the affinity of TRPC5 for lipids because tryptophan generally exhibits the most favorable interactions with lipids and is often located at the water–lipid bilayer interface rather than deeply buried in the membrane [40]. We found that the mutation G606W restored W577A channel activity induced by depolarizing voltage in the extracellular solution containing Gd^3+^, and the W577A/G606W-mediated responses were further potentiated by LPC treatment (Fig. 3B, C, F and G). The single G606W mutation generated a gain-of-function phenotype with a left-shifted voltage dependence of activation compared to wild-type channels (*V*_50_ = 116.0 ± 5.0 mV, *P* ≤ 0.001; *z* = 0.76 ± 0.4 e_0_; *n* = 6; *P* = 0.960; two-tailed *t*-test; Supplementary Fig. S5A-C). LPC potentiated the G606W-mediated voltage-induced currents less than those of the wild-type channels, and importantly, only small if any fluctuations at highly positive potentials were observed (Supplementary Fig. S5D–F). EA (30 nM) neither produced any responses nor sensitized LPC-induced currents in this mutant over the time interval studied (Supplementary Fig. S5D-I). These results suggest that tryptophan at position 606 disrupts the binding site for EA and acts to stabilize the open state of the channel either by increasing the affinity for resident lipids or by contacting the opposite W577, thereby further stabilizing the pore domain during voltage-dependent gating.

**Fig. 4:**
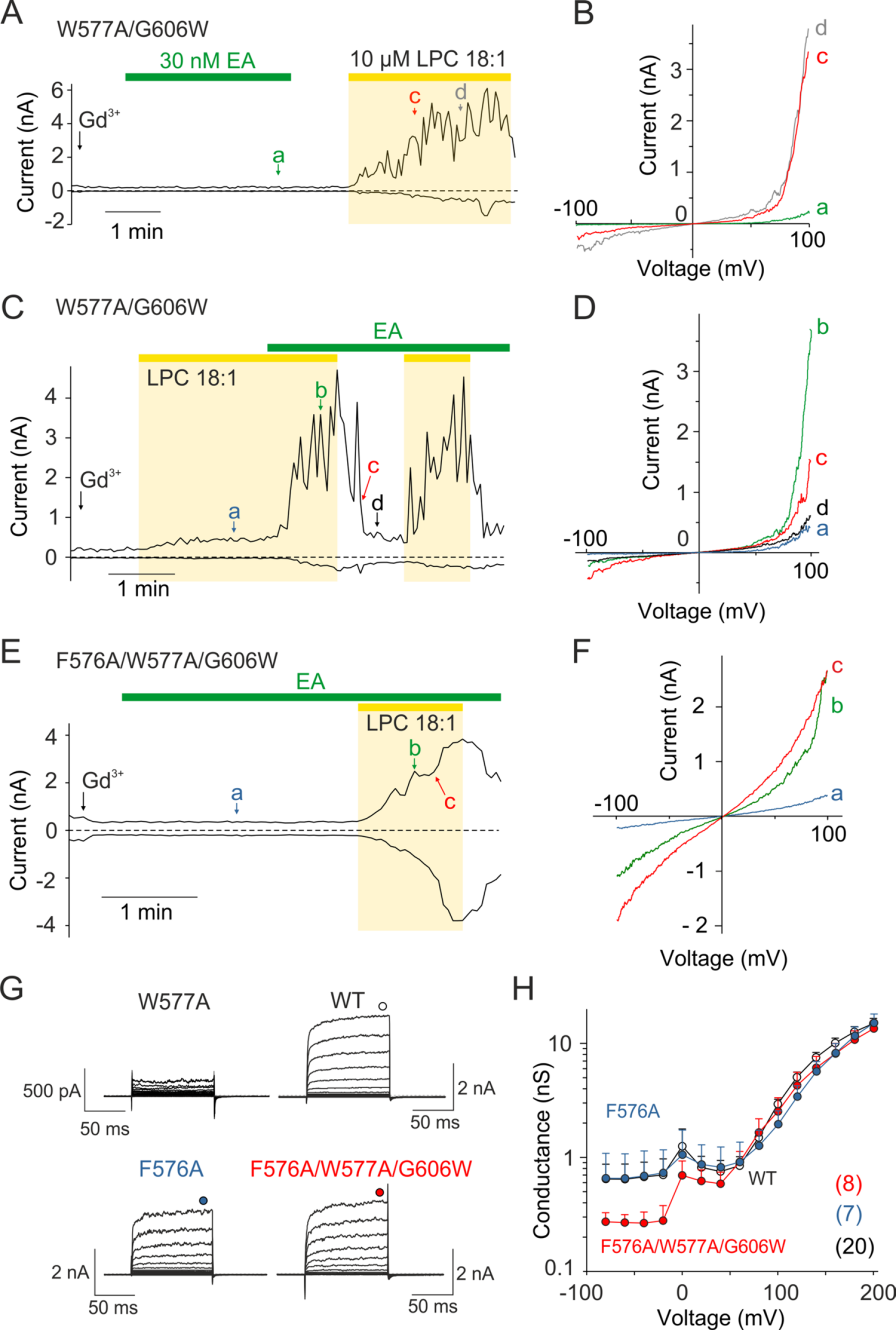
The xanthine-binding pocket is involved in the LPC-induced activation of TRPC5. **A**,** C** Representative time course of currents induced by LPC 18:1 (10 µM) in the double mutant W577A/G606W TRPC5 channels recorded in the absence or presence of (−)-englerin A (EA; 30 nM). A ramp pulse protocol as shown in Fig. 1A. Amplitudes were measured at − 100 mV and + 100 mV and plotted as a function of time. **B**,** D** The current–voltage relations at the time points indicated by the colored letters in panel A and C. **E** Representative time course of currents measured in the presence of (−)-englerin A (EA; 30 nM) and LPC 18:1 (10 µM) in the triple mutant F576A/W577A/G606W channels. A ramp pulse protocol as shown in Fig. 1A was applied and amplitudes measured at − 100 mV and + 100 mV were plotted as a function of time. **F** The current–voltage relations at the time points indicated by the colored letters in panel E. **G** Average current traces in response to a voltage step protocol (from − 80 to + 200 mV; as shown in Fig. 1I) recorded from cells expressing W577A (*n* = 7), wild-type (WT; *n* = 20), F576A (*n* = 7), or triple mutant F576A/W577A/G606W (*n* = 8) TRPC5-channels as indicated. The currents were recorded in the presence of 10 μM Gd^3+^. **H** The average conductance-voltage plots from measurements as in E for indicated constructs obtained in extracellular control solution containing Gd^3+^. Steady-state currents were measured at the end of the pulses (indicated by circles above the records in G). Number of biological replicates for each condition is indicated in parentheses. Data are mean + SEM

The W577A/G606W mutant channels exhibited a very interesting functional response. They were sensitive to LPC similar to the wild-type channels (Supplementary Fig. S7K) but insensitive to EA (Fig. 4A and B). The application of EA together with LPC induced large responses and the fluctuations in the amplitude of the outward currents at positive potentials increased very dramatically (Fig. 4C and D). This contrasts strongly with the currents of the wild-type channels, for which the fluctuations completely disappeared upon prolonged exposure to EA (see Fig. 2E), and with G606W, that was completely insensitive to EA (Supplementary Fig. S5D-I). These findings indicate that LPC and EA do not act additively and that LPC may bind in a site that allosterically couples to the EA binding site. The effect of EA on the W577A/G606W channels (Fig. 4C) was strikingly similar to that of Pico145 applied in the presence of LPC in wild-type channels (Fig. 2A), suggesting significant involvement of the xanthine-binding pocket in the LPC-induced activation. Similar to G606W, the F576A/W577A/G606W triple mutant was completely unresponsive to EA (Fig. 4E and F), confirming that the presence of F576 is essential for EA activation [14] and that the large aromatic residue at position 606 does not compensate for it. The triple mutant retained sensitivity to LPC (Supplementary Fig. S7K) and exhibited normal current–voltage characteristics similar to wild-type TRPC5 (Fig. 4G), with only a decrease in basal currents at hyperpolarizing potentials (Fig. 4H). Interestingly, the F576A single mutation did not abolish the voltage sensitivity of the channel (in contrast to the W577A mutation), although it produced a slight rightward shift in current–voltage characteristics as compared with the wild-type channel (Fig. 4H). These data suggest that tryptophan introduced at position 606 or the presence of LPC can functionally compensate for the lack of tryptophan at position 577 in the activation of TRPC5 by voltage.

To further explore the involvement of the critical residues W577 and G606 in a more physiologically relevant mode of TRPC5 activation, we co-expressed TRPC5 constructs with the plasmid-encoding human muscarinic receptor type 3 (M3) in HEK293T cells and tested the G606W, W577A and W577A/G606W mutants for sensitivity to carbachol (Supplementary Fig. S6). The wild-type and G606W channels exhibited robust responses to 100 µM carbachol. Upon addition of 10 µM LPC, the carbachol-induced responses mediated by wild-type channels increased slightly at both negative and positive potentials and then desensitized. In contrast, the W577A and W577A/G606W mutant channels showed only weak responses to carbachol, and the addition of LPC inhibited the inward currents at negative potentials and increased outward currents at positive potentials (Supplementary Fig. S6H-K). These results demonstrate that, in addition to the important role of W577 and G606 in LPC-dependent regulation of TRPC5, the two residues are also involved in the activation by a signal downstream of the G-protein-coupled receptor-G_q/11_ phospholipase C pathway.

### The L1 lipid-recognition site is not involved in LPC-mediated TRPC5 activation

The results described above suggest that the highly conserved lipid-recognition site L2 in the pore domain is specifically involved in the effects of LPC on TRPC5. Current structural studies on TRPC channels reveal several different binding sites for lipids [14, 16, 17, 41–43]. One of these, a lipid-binding fenestration designated L1 in TRPC3 [22, 44], is located within the voltage-sensor like domain, near the S2-S3 linker, S4-S5 linker and TRP helix. In TRPC5, the homologous L1 site is most likely occupied by phosphatidylinositol 4,5-bisphosphate (PIP_2_), which is essential for channel activation [45], and is coordinated by positively charged residues K228, K232, K299, R512 and K645, whereas its fatty acyl chains extensively contact two tryptophan residues W434 and W435 in the S2-S3 linker [42]. To explore the possible involvement of the L1 site in LPC-induced activation, we measured currents through the R512A and W434A/W435A mutants and found that both constructs exhibit no lower responsiveness to LPC compared with wild-type channels (Supplementary Fig. S7A-E and K). It is therefore unlikely that the L1 lipid coordination site is responsible for LPC-induced TRPC5 activation.

### Voltage does not act through the activation pathway for protons and probably does not involve the pore domain

Our results indicate that the sensitizing effect of LPC on TRPC5 is voltage dependent. However, the molecular basis of TRPC5 activation by voltage is unknown. To understand how LPC may interact with voltage, we considered different voltage sensitivity mechanisms that are known for other voltage-sensitive channels [46]. First, we neutralized the arginine at position 492, which is the only basic residue located in the transmembrane S4 helix (Supplementary Fig. S7A), and measured responses to voltage steps. The R492Q mutant did not exhibit any significant activity to strong (+ 200 mV) depolarization (Supplementary Fig. S7F). Because R492 is oriented to the interior of the sensor cavity where it contributes to the coordination of the calcium ion, its neutralization is expected to weaken this interaction. To overcome this weakening and test the involvement intracellular Ca^2+^, we increased the concentration of free Ca^2+^ in the recording pipette to 100 µM. Under these conditions, the R492Q mutant was still completely insensitive to voltage in control extracellular solution but responded readily to LPC and also to EA (Supplementary Fig. S7F–J). This result suggests that R492 is not crucial for the dominant observed effects of LPC. On the other hand, further experiments would be necessary to determine whether this residue may be part of the putative TRPC5 voltage sensor.

We next considered an “unorthodox mechanism” of voltage-dependent activation that has been proposed for the related TRPV1 channel and involves acidic outer pore residues engaged in modulation by extracellular pH [26, 46]. Because the potentiating effects of protons on TRPC5 are very similar to those on TRPV1, we tested the hypothesis that the voltage sensitivity of TRPC5 may underlie the mode of proton activation, thereby influencing the upper gate in the selectivity filter. Comparison of voltage-dependent TRPC5-mediated currents in control solution containing 10 µM Gd^3+^ and upon addition of protons (pH 6.5) showed that deep hyperpolarization (to − 260 mV) cannot counteract proton activation (Supplementary Fig. S8A–C). That is, the voltage does not act through the activation pathway for protons. The glutamate residues responsible for proton sensitivity of TRPC5 (E543 and E595) are conserved in the related TRPC4, and the L2 lipid-binding site between the two channels is also fully conserved, except for the rather conservative substitution of V579 and L583 for isoleucines in TRPC4 (Supplementary Fig. S8A). In our hands, TRPC4 showed no response to the voltage step protocol (Supplementary Fig. S7D and E). Moreover, the V579I/L583I mutant of TRPC5 exhibited wild-type phenotype (Supplementary Fig. S8F and G), further underscoring that the voltage sensitivity of TRPC5 likely comes from a distinct region than from the acidic outer pore residues.

### Molecular dynamics simulations of TRPC5 activation by depolarizing voltage indicate involvement of the L2 lipid-recognition site

**Fig. 5:**
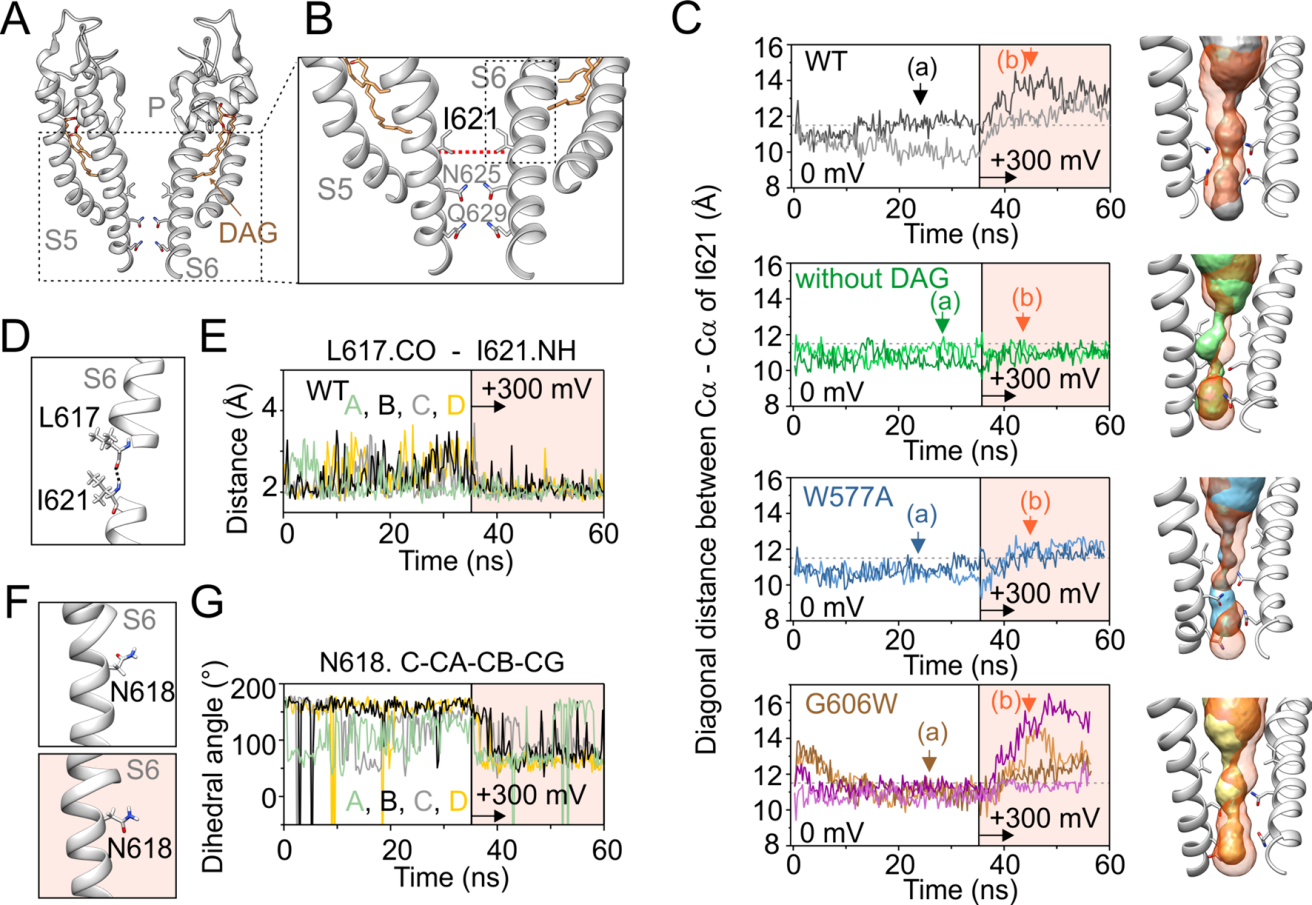
Molecular dynamics simulations indicate that changes in the L2 lipid-binding site influence the lower gate of TRPC5 during depolarization. **A** Side view of the pore domain structure of two opposite subunits of human TRPC5 (PDB ID: 7E4T) with indicated residues forming the lower gate restriction (I621, N625, Q629). **B** Zoomed-in view of the lower pore region with the distance between the backbone Cα of two opposite residues I621 indicated. **C** Instantaneous distance measured diagonally between the backbone Cα of I621 in S6 of chains A and C (darker colored line), and chains B and D (lighter colored line), plotted as a function of simulation time for wild-type structure (7E4T), the structure without YZY lipid (DAG), and for the W577A and G606W mutants. Two independent simulations are shown for G606W. The colored (light orange) area indicates the time when depolarization at + 300 mV was applied. The horizontal dashed line denotes the diagonal distance between the backbone Cα of isoleucines 621 measured from the initial structure (11.51 Å). Right, side view of the profiles of the pore domain of two diagonally-arranged subunits of the respective constructs shown left with indicated residues in the lower gate I621, N625 and Q629. Instantaneous tunnels were calculated by Caver Analyst at points (a; colored) and (b; light orange) depicted by arrows in the plots on left. **D** Enlarged view of S6 with the hydrogen bond between the backbone CO group of L617 and NH group of I621 outlined. **E** Distance between the backbone CO group of L617 and NH group of I621, plotted as a function of simulation time for the chains A, B, C and D of the wild-type structure (7E4T). The colored area indicates the time when depolarization at + 300 mV was applied. **F** Zoomed-in view of the side-chain orientation of the residue N618 at control condition (wild-type structure without applied depolarization voltage) and at + 300 mV (colored area). **G** Changes in the dihedral angle values for the side-chain of N618 (C-CA-CB-CG), plotted as a function of simulation time for the chains A, B, C and D of the wild-type structure (7E4T)

To further understand the dynamic changes underlying voltage- and LPC-dependent activation of TRPC5, we performed a series of molecular dynamics (MD) simulations (Fig. 5 and Supplementary Fig. S9). Using the apo structure of the human TRPC5 (7E4T), we built four MD simulation systems: (i) wild-type TRPC5, (ii) the channel without the presence of the DAG (YZY) molecule in the L2 lipid-binding site, (iii) the W577A mutant and (iv) the G606W mutant. The structures were embedded into the phosphatidyl-ethanolamine (PEA) membrane and 36-ns initial simulations with default parameters were run using the YASARA Structure program. A depolarizing voltage of + 300 mV was then applied. The application of + 200 mV led to entirely analogous changes as at + 300 mV (Supplementary Fig. S10), so we chose the higher potential to maximize the observed effects. In the wild-type channel, depolarizing voltage consistently led to an increase in the distances between the diagonally opposed residues I621 that form the lower hydrophobic gate (Fig. 5A–C, Supplementary Fig. S9 and Movie 1). This incomplete opening of the lower gate was apparently associated with stabilization of the hydrogen bonds between the backbone CO group of L617 and NH group of I621 (and partially between L613 and N618), indicating that complementary charge interactions in a depolarized membrane environment can represent a driving force for conformational changes within the S6 helix (Fig. 5D–G). Changes in the distances between I621 were smaller during depolarization in the W577A mutant, and none were observed in the channel lacking DAG (Fig. 5C). In contrast, the G606W mutant exhibited a permeation pathway that significantly widened near I621, N625 and Q629 upon depolarization, and this widening was often asymmetric.

## Discussion

In the present study, we used Gd^3+^ in extracellular solution, which adds a certain complexity to the interpretation of some of our results. We demonstrate that LPC-induced currents obtained in the absence of Gd^3+^ did not exhibit the clearly doubly rectifying current–voltage relationships typical of TRPC5 (see Supplementary Figure S1A–E). LPC exerts many different effects on a broad range of receptors and ion channels [1–3], and identifying all the endogenous targets contributing to LPC responses in HEK293T cells would be a challenging task. LPC activates, among others, calcium-permeable acid sensing ion channels at physiological pH [2], which can be blocked by Gd^3+^ [47]. In this regard, Gd^3+^ has a convenient general discriminatory capability because it blocks non-selective cation channels and other Ca^2+^-permeable channels while promoting TRPC5 activity. Gd^3+^ was constantly present in our experiments at a concentration of 10 µM that did not consistently activate the channels at membrane potentials from − 100 mV to + 100 mV (please see zoomed view in Fig. 1C and corresponding legend). It should be noted that 10 µM Gd^3+^ was sufficient to activate TRPC5 in some studies, but these experiments were mostly performed in the presence of histamine or carbachol in cells that also contained a G_q/11_-protein-coupled receptor (histamine or muscarinic) [25, 48]. Although Gd^3+^ allosterically contributes to the activation mechanism of LPC, it interacts with the receptor at the same sites as protons (i.e. E543 and E595)[25, 26] and our results show that depolarizing voltage likely does not act via the activation pathway for protons (Supplementary Figure S8). Therefore, we do not consider the presence of low (10 µM) concentrations of Gd^3+^ to be crucial for voltage-dependent activation of the channel and our proposed mechanism of action of LPC is valid under these conditions.

In order to maintain conditions as similar as possible to those described by Flemming et al. (2006) [7], we used ATP in the recording pipette solution (2 mM). Recently, ATP has been found to inhibit GTPγS-induced TRPC5 currents to basal levels (but not below) at both negative and positive membrane potentials [49]. The effects described in our study are voltage dependent and therefore we do not attribute them to the relieved ATP-dependent inhibition of TRPC5.

The results from our MD simulations are consistent with the experimental data and indicate that, under certain conditions such as membrane depolarization, the residues forming the lower hydrophobic gate of TRPC5 can be affected by mutations in the vicinity of the selectivity filter or by removal of resident lipids in the L2 lipid recognition site. So how can LPC, which presumably interacts with the upper pore region, open the ion channel or reduce the activation energy for its opening? The molecular mechanism of TRP channel activation often involves the rotation of a conserved asparagine in S6 from a position facing the S4-S5 linker toward the pore [50–53]. This rotation is facilitated by the π-bulge in the S6 helix, which is evolutionarily conserved across the entire TRP family. Indeed, the structures of the different TRP channels show that the orientation of the residue corresponding to N618 in TRPC5 relative to the pore can vary by up to 180° from structure to structure [50]. Inward rotation of asparagine (Fig. 5F, G) is associated with dehydration of the four peripheral cavities located between each S6 and the S4-S5 linker and with hydration of the pore that facilitates ion permeation [50]. The degree of TRPC5 opening by depolarizing voltage (Ile621 C_α_-C_α_ distance ~ 12–13 Å) that we observed in our MD simulations is mostly similar to that observed, for example, in the cryo-EM structure of TRPV1 (PDB ID: 7LPE [54]—Ile679 C_α_-C_α_ distance 13.2 Å). Only in the case of the G606W mutant we observed an apparently full (albeit asymmetric) opening above the ~ 14–15 Å level. This corresponds, for example, to the open structures of TRPV5 (14.9 Å; PDB ID: 6DMU [55]) or TRPM5 (14.1 Å; PDB ID: 7MBS [56]). Comparison of the aforementioned experimental structures of TRPV1 (PDB ID: 7LPE) vs. TRPV5, TRPM5 (PDB ID: 6DMU, 7MBS) shows that the N618 side chain needs to be wedged between the I621 side chains to fully open the lower hydrophobic gate. This is exactly what happened in the case of one of the N618 side chains in the G606W system (Fig. 5C). In fact, the spontaneous, symmetrical and full opening of the lower hydrophobic gate for large and complex TRP ion channels is mostly beyond the scope of capabilities of contemporary hardware resources, including specialized supercomputers [57].

In conclusion, we report for the first time that lysophosphatidylcholine 18:1 regulates TRPC5 channel by potentiating the voltage-dependent mode of its activation, and that this process involves conserved residues within the lateral fenestration of the pore domain. Because the outer pore region of TRPC5 is the most likely binding site for DAG, which, despite its potential role as an activator, may be also present in the inactive conformation of the channel [17], LPC may act through DAG, or substitute it, to promote channel activation. The inhomogeneous embedding of LPC in the membrane, and thus within the microenvironment around the L2 lipid coordination site, may then explain the observed amplitude fluctuations upon strong depolarization, when electrostatic changes occur in the charged regions of the channel and polar heads of membrane lipids. If future structural studies confirm this hypothesis, it will provide a basis for the possibility of TRPC5 regulation under a range of pathophysiological conditions.

In addition, and perhaps just as importantly, the data we present have an intriguing implication with significant translational potential: we have discovered a TRPC5 mutant (G606W) that can form functional channels (responsive to activation by the physiological compound carbachol) but is insensitive to the specific agonist (−)-englerin A. The concept of using compound-insensitive variants for genetic rescue experiments is currently considered the most advanced method for preclinical target validation [58]. Therefore, our findings could potentially be used to develop a strategy for dissecting on- and off-target effects of (−)-englerin A in future functional studies.

## Materials and methods

### Cell culture and transfection

HEK293T cells (ATCC, Manassas, VA, USA) were cultured in Opti-MEM I medium (Thermo Fisher Scientific, Waltham, MA, USA) supplemented with 5% fetal bovine serum (PAN-Biotech, Germany). F11 cells (The European Collection of Authenticated Cell Cultures, ECACC 08062601, Porton Down, UK) were cultured in Dulbecco’s Modified Eagle’s Medium supplemented with 2 mM glutamine and 10% fetal bovine serum. One to three days before transfection, cells were plated onto 24-well plates in 0.5 ml of medium and became 60% confluent on the day of transfection. HEK293T cells were transiently co-transfected with 300 ng of plasmid encoding wild-type or mutant human TRPC5 (in the pCMV6-XL5 vector, Origene, Rockville, MD, USA) and with 200 ng of plasmid encoding GFP (Takara, Osaka, Japan) using the magnet-assisted transfection technique (IBA GmbH, Goettingen, Germany), and then plated on poly-L-lysine-coated glass coverslips. The plasmid-encoding human muscarinic receptor type 3 (M3) (in the pcDNA3.1 vector, Missouri S&T cDNA Resource Center, Rolla, MO, USA) was co-transfected (300 ng of cDNA) for the experiments with carbachol. F11 cells were transiently co-transfected with 600 ng of cDNA plasmid-encoding wild-type human TRPC5 and 400 ng of GFP plasmid (Takara, Osaka, Japan) with the use of Lipofectamin 2000 (Invitrogen, Carlsbad, CA, USA). The cells were used 48–72 h after transfection. The wild-type channel was regularly tested in the same batch as the mutant. The mutants were generated by PCR using a QuikChange II XL Site-Directed Mutagenesis Kit (Agilent Technologies, Santa Clara, CA, USA) and confirmed by DNA sequencing (Eurofins Genomics, Ebersberg, Germany).

### Electrophysiology

Whole-cell currents were recorded by employing an Axopatch 200B amplifier and the software pCLAMP version 10.6 (Molecular Devices, San Jose, CA, USA). Patch electrodes were pulled from borosilicate glass capillaries with a 1.5-mm outer diameter (Science Products GmbH, Germany) using a horizontal puller (P-87, Sutter Instrument Co., CA, USA) and heat-polished (MF-83, Narishige, Tokyo, Japan) to have a resistance of 3–5 MΩ when filled with the appropriate solution. Series resistance was compensated by at least 60%. The extracellular bath solution for whole-cell measurements contained (in mM): 160 NaCl, 2.5 KCl, 1 CaCl_2_, 2 MgCl_2_, and 10 HEPES (4-(2-Hydroxyethyl)piperazine-1-ethanesulfonic acid) and 10 glucose; adjusted to pH 7.3 or 6.5 (for indicated measurements) with NaOH, 310 mosmol·l^−1^. Intracellular solution containing (in mM): 145 CsCl, 3 CaCl_2_, 2 MgATP, 10 HEPES, and 5 EGTA (adjusted to pH 7.3 with CsOH, 300 mosmol·l^-1^) was used unless the usage of low-buffer internal solution is noted, which contained (in mM): 145 CsCl, 10 EGTA, 10.24 CaCl_2_ (corresponding to 100 µM free Ca^2+^), 10 HEPES, and 2 MgATP (adjusted to pH 7.3 with CsOH and to 290 mosmol·l^−1^). The liquid-junction potential was calculated to be + 4.9 mV using Clampex 10.4 software; data were not corrected for this offset. Data were low-pass filtered at 2 kHz through the built-in 8-pole Bessel filter and digitized at 5–10 kHz with a Digidata 1550B analog-to-digital converter equipped with HumSilencer and controlled by Clampex 10.6 (Molecular Devices, San Jose, CA, USA). Only one recording was performed on any one coverslip of cells to ensure that recordings were made from cells not previously exposed to chemical stimuli. The experiments were performed at room temperature (23–25 °C). Data were obtained from at least three independent transfections.

LPC 18:1 (Avanti Polar Lipids, Alabaster, AL, USA) was prepared at desired concentration in extracellular bath solution immediately before use from a 48 mM stock solution in ethanol (stored in the − 80 °C freezer) and was used no longer than 2 h after preparation. Final ethanol concentration was < 0.0001%. (−)-englerin A (Phytolab GmbH & Co. KG, Vestenbergsgreuth, Germany) and Pico145 (MedChemExpress Inc., Monmouth Junction, NJ, USA) were dissolved in dimethyl sulfoxide (DMSO) to prepare their 1 mM and 0.1 mM stock solutions, respectively, aliquots of which were stored at − 80 °C. Further dilutions to the required concentration were made with extracellular bath solution and the final concentration of DMSO did not exceed 0.1%. Before each experiment, the gravity-driven PTFE/glass multi-barrel perfusion system was saturated by 5–10 ml LPC 18:1, (−)-englerin A or Pico145 at the concentrations used. The efficacy of 10 µM LPC was checked at the beginning of each experimental day using the wild-type TRPC5. All other chemicals were purchased from Sigma-Aldrich (Merck Life Science, Prague, Czech Republic).

### Molecular modeling

The apo state structure of human TRPC5 was retrieved from the RCSB protein database (PDB ID: 7E4T). Molecular dynamics (MD) simulations were performed using the “em_runclean.mcr “ and then “md_runmembranefast.mcr” macro within YASARA Structure (version 22.9.24; YASARA Biosciences). The following default parameters were used: memextension = 15, waterextension = 10, square = 1, ions = 'Na,Cl,0.9', ForceField AMBER14, temperature = '298 K' and pressure = 1. The structures were embedded into the phosphatidyl-ethanolamine (PEA; 1-palmitoyl, 2-oleoyl by default) membrane. We used PEA because it is the most stable membrane lipid and other membrane compositions we examined (phosphatidyl-choline: phosphatidyl-ethanolamine: cholesterol, 2:1:1, and phosphatidyl-choline: cholesterol, 1:1) gave similar results. The trajectories were analyzed using VMD (version 1.9.4) [59] and Chimera (version 1.17) [60]. Mutations were introduced by the „Swap” command and diacylglycerol (YZY lipid) was removed by the “Delete” command using YASARA. After initial equilibration and a 36-ns-long simulation under control conditions, the membrane potential was stepped by applying a constant electrostatic field with the < AddESF > command to mimic a membrane potential within the appropriate context of the macro „md_runmembrane.mcr”. Each simulation was repeated at least three times from the beginning. After 20–30 ns MD simulation at + 300 mV, water molecules frequently began to penetrate the membrane bilayer. However, the effects of depolarization on the protein were reversible upon hyperpolarization to − 300 mV (see Supplementary Fig. S9C) and fully reproducible upon repeated simulations (see Supplementary Fig. S9B). The membrane instability was not observed at + 200 mV. The simulation approach was further validated using the closed state structure of zebrafish TRPM5 channel (PDB ID: 7MBR; Supplementary Fig. S11). Permeation pathways were estimated using CAVER Analyst (version 2.0) [61]. The AutoDock Vina tool was used to predict the possible binding modes of LPC to the xanthine binding site of TRPC5 (7E4T) and its mutants. LPC was set as flexible for the docking.

### Statistics

Data were analyzed using Clampfit 11 (Molecular Devices, San Jose, CA, USA), SigmaPlot 10 (Systat Software Inc., San Jose, CA, USA) and OriginPro 2021 (OriginLab Corporation, Northampton, MA, USA). Voltage-dependent gating parameters were estimated from steady state conductance–voltage (*G*/*V*) relationships obtained at the end of 100-ms voltage steps by fitting the conductance *G* = I/(*V* − *V*_rev_) as a function of the test potential *V* to the Boltzman equation:

*G* = ((*G*_max_ − *G*_min_)/(1 + exp[− *zF*(*V* − *V*_50_)/*RT*])) + *G*_min_, where *z* is the apparent number of gating charges involved in channel opening (in elementary charge units: *e*_o_ = 1.6 × 10^−19^ C), *V*_50_ is the half-activation voltage, *G*_min_ and *G*_max_ are the minimum and maximum whole-cell conductance, *V*_rev_ is the reversal potential, and *F*, *R*, and *T* have their usual thermodynamic meaning. Concentration–response curves were fitted to the Hill equation *I*/*I*_max_ = 1/[1 + (*EC*_50_/*C*)^h^], where *EC*_50_ is the half-maximal effective concentration, *C* is the drug concentration, *h* is the Hill coefficient, and *I*_max_ is the maximum current. Throughout, average data are presented as means ± standard error of the mean, SEM, or as a median, range, and interquartile range as appropriate. Statistical significance was calculated using Student’s *t* test, Mann–Whitney rank-sum, or one-way analysis of variance followed by the non-parametric Dunn’s test, as appropriate. Differences were considered significant at *P* < 0.05.

### Supplementary Information

Below is the link to the electronic supplementary material.

Supplementary file1 (DOCX 4154 KB)

Supplementary file2 (MP4 20780 KB)

## Data Availability

All data generated or analysed during this study and its supplementary information files are available from the corresponding author on reasonable request.
