# Supplementary material for: Functional determinants of lysophospholipid- and voltage-dependent regulation of TRPC5 channel
Source: Cell Mol Life Sci. 2024 Aug 29;81(1):374. doi: 10.1007/s00018-024-05417-7 (PMC11362415; doi:10.1007/s00018-024-05417-7)
Supplement: Supplementary file 1 — Supplementary file1 (DOCX 4154 KB) [file 18_2024_5417_MOESM1_ESM.docx]

# Supplementary Information

Functional determinants of lysophospholipid- and voltage-dependent regulation of TRPC5 channel

Alexandra Ptakova^1,2^, Lucie Zimova^1^, Ivan Barvik^3^, Robin S. Bon^4^ and Viktorie Vlachova^1,*^

**Supplementary Figure S1** | Main characteristics of LPC-induced responses measured in human embryonic kidney 293T cells transfected with human TRPC5

**Supplementary Figure S2** | LPC potentiates the voltage-dependent mode of activation of TRPC5

**Supplementary Figure S3** | Pico145 inhibits TRPC5-mediated currents induced by (-)-englerin applied together with LPC 18:1. LPC activates non-TRPC5 mediated currents in F11 cells

**Supplementary Figure S4** | Molecular docking suggesting a possible interaction between the L2 lipid recognition (xanthine-binding) site and LPC

**Supplementary Figure S5** | Mutation G606W produces gain-of-function phenotype and renders TRPC5 insensitive to (-)-englerin A

**Supplementary Figure S6** | Effect of carbachol on critical TRPC5 constructs

**Supplementary Figure S7** | Mutations of sites potentially involved in voltage-dependent activation of TRPC5 do not eliminate LPC sensitivity

**Supplementary Figure S8** | Voltage does not act through the activation pathway for protons

**Supplementary Figure S9** | Molecular dynamics simulations of TRPC5 during depolarization and hyperpolarization

**Supplementary Figure S10** | Molecular dynamics simulations at +200 mV

**Supplementary Figure S11**| Molecular dynamics simulations of TRPM5 during depolarization

**Supplementary Movie 1** | Voltage-induced changes of the pore domain of human TRPC5 channel


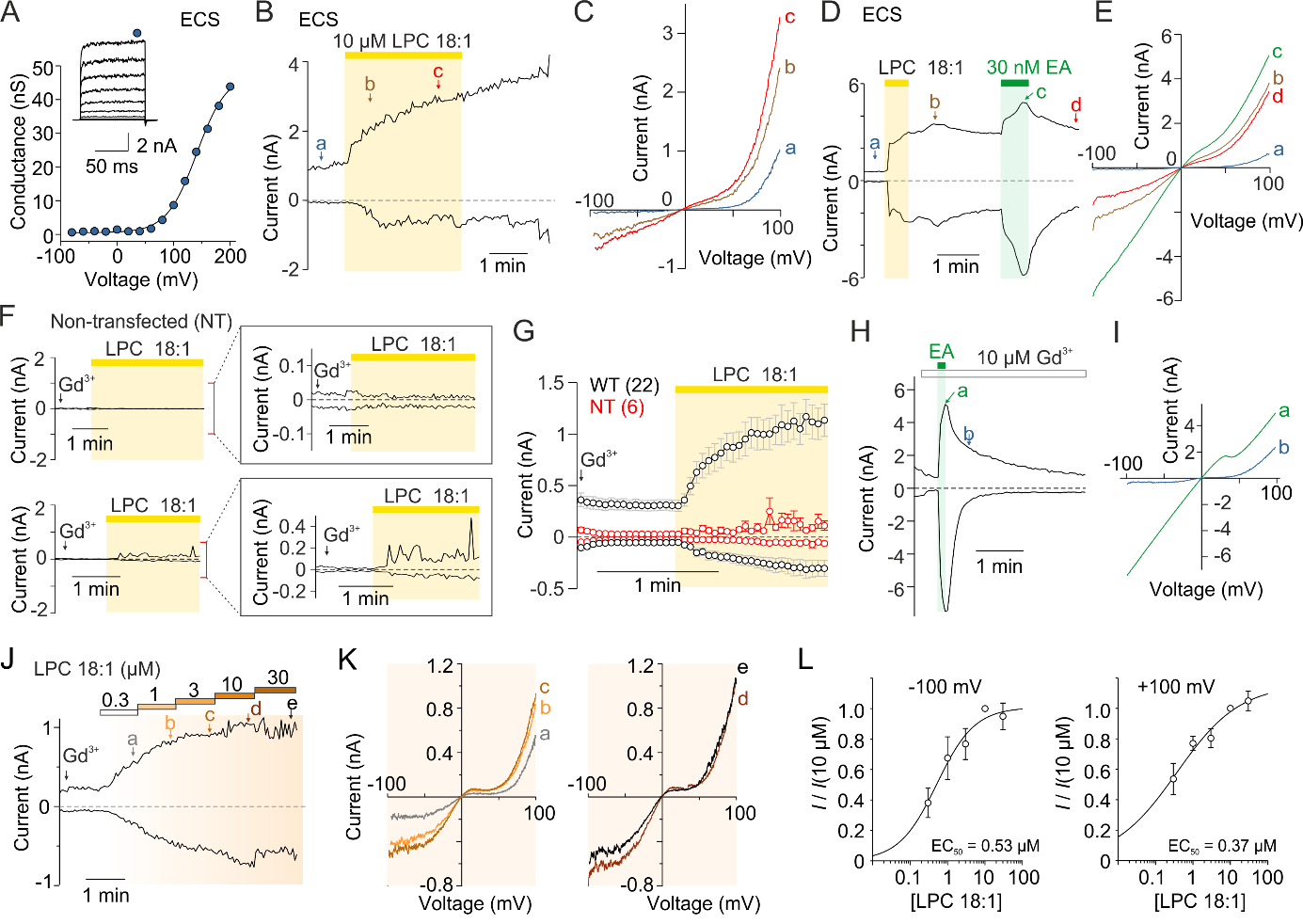


**Supplementary Figure S1** **|** **Main characteristics of LPC-induced responses measured in human embryonic kidney 293T cells transfected with human TRPC5**

(**A**) The conductance-voltage plot obtained from current traces recorded from a TRPC5-expressing HEK293T cell exposed to 100-ms voltage steps from -80 to +200 mV (inset) measured in extracellular control solution (ECS) without the presence of Gd^3+^. (**B**) Representative whole-cell currents recorded from the same cell as shown in A, measured in ECS. Application of 10 µM LPC (LPC 18:1) is indicated by horizontal bar above the record. A ramp pulse from -100 mV to +100 mV from a holding potential of 0 mV was periodically applied every 3 seconds for 500 ms (protocol shown in Fig. 1A). Amplitudes were measured at -100 mV and +100 mV and plotted as a function of time. (**C**) The current-voltage relations at the time points indicated by the letters in panel B. (**D**) Representative whole-cell currents recorded from another TRPC5-expressing HEK293T cell. Applications of 10 µM LPC (LPC 18:1) and 30 nM (-)-englerin A (EA) are indicated by horizontal bars above the record. (**E**) The current-voltage relations at the time points indicated by the letters in panel D. (**F**) Representative currents recorded from two non-transfected control HEK293T cells in extracellular solution containing 10 µM Gd^3+^ (indicated by vertical arrow), measured at -100 mV and +100 mV. The traces shown on the left are at the commonly used scale of membrane currents and plotted at the enlarged scale on the right. (**G**) Time course of average whole-cell currents measured in the absence and presence of 10 µM LPC 18:1 in non-transfected and TRPC5-expressing cells. Error bars represent ± SEM, number of cells is indicated in parentheses. (**H**) Representative whole-cell currents induced by 30 nM (-)-englerin A (EA) recorded from a TRPC5-expressing HEK293T cell in extracellular solution containing 10 µM Gd^3+^. A ramp pulse from -100 mV to +100 mV from a holding potential of 0 mV was periodically applied every 3 seconds for 500 ms (protocol shown in Fig. 1A). Amplitudes were measured at -100 mV and +100 mV and plotted as a function of time. (**I**) The current-voltage relations at the time points indicated by the letters in panel H. (**J**) Representative TRPC5 currents induced by LPC 18:1 at indicated concentrations measured at -100 mV and +100 mV. (**K**) The current-voltage relations at the time points indicated by the letters in panel J. (**L**) Dose-response data for TRPC5-mediated currents measured at -100 mV (left) and +100 mV (right) as shown in panel J, normalized at 10 µM LPC.


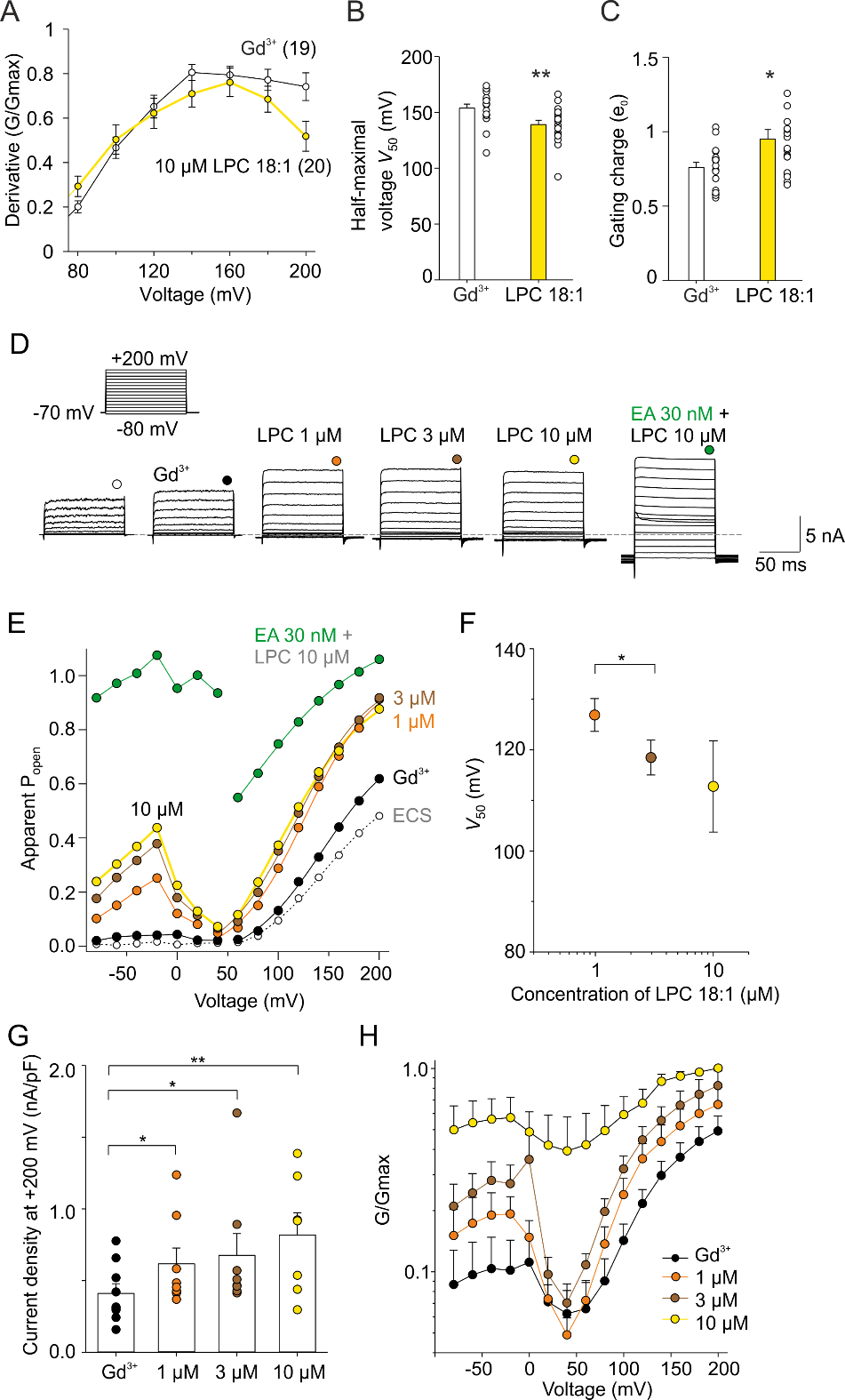


**Supplementary Figure S2** **|** **LPC potentiates the voltage-dependent mode of activation of TRPC5**

(**A-C**) The average normalized derivative of conductances plotted as a function of voltage, measured from TRPC5-expressing cells in extracellular control solution containing 10 µM Gd^3+^ (black line and empty circles) and in the presence of 10 µM LPC 18:1 (yellow line and yellow circles) recorded as in Fig. 1I and J. The peak of the plot corresponds to the inflection point of the Boltzmann relationship, indicating that the estimation of the values of the half-maximal activation voltage (*V*_50_) and the steepness (gating charge; *z*) can be reliably estimated over the interval studied. These values are compared in panels B (***P* = 0.002) and C (**P* = 0.025; two-tailed paired *t*-test). Number of biological replicates for each condition is indicated in A in parentheses. The point plot in A shows the mean ± SEM, the bar graphs in B and C show the mean + SEM. (**D**) Representative whole-cell current traces elicited by a voltage step protocol in the absence (ECS) or presence of Gd^3+^ (10 µM), and Gd^3+^ with LPC 18:1 or LPC 18:1 together with (-)-englerin (30 nM, EA). (**E**) Normalized steady-state maximal conductance curves obtained from current traces shown in panel D. Lines represent Boltzmann fit to the data over the interval from +60 mV to +200 mV. At negative membrane potentials, the lines connect the points. (**F**) Half-maximal voltage *V*_50_ as a function of LPC 18:1 concentration (127.0 ± 3.2 mV at 1 µM, 118.6 ± 3.5 mV at 3 µM and 112.8 ± 9.0 mV at 10 µM). The apparent number of gating charges (*z*) decreased from 0.83 ± 0.11 e_0_ to 0.72 ± 0.08 e_0_, 0.68 ± 0.03 e_0_ and 0.67 ± 0.09 e_0_ at 1, 3 and 10 µM LPC, respectively (paired *t*-test, **P* < 0.05). Error bars represent ± SEM (*n* = 8). In the presence of 10 µM LPC, the TRPC5 currents desensitized and their estimated *V*_50_ values were not significantly different from currents measured in the presence of 1 µM LPC (*P* = 0.093), but still were significantly different from the values of *V*_50_ estimated for control currents measured in the presence of 10 µM Gd^3+^ (paired *t*-test, **P* < 0.05). (**G**) Maximal current density at +200 mV during application of 10 µM Gd^3+^ and LPC (1 µM, 3 µM and 10 µM), paired *t*-test, **P* < 0.05, ***P* < 0.01. (**H**) Average steady-state maximal conductance curves obtained from current traces as shown in D, normalized to maximal conductance at 10 µM LPC. Error bars represent ± SEM (*n* = 6). The lines connecting data points have no theoretical meaning.


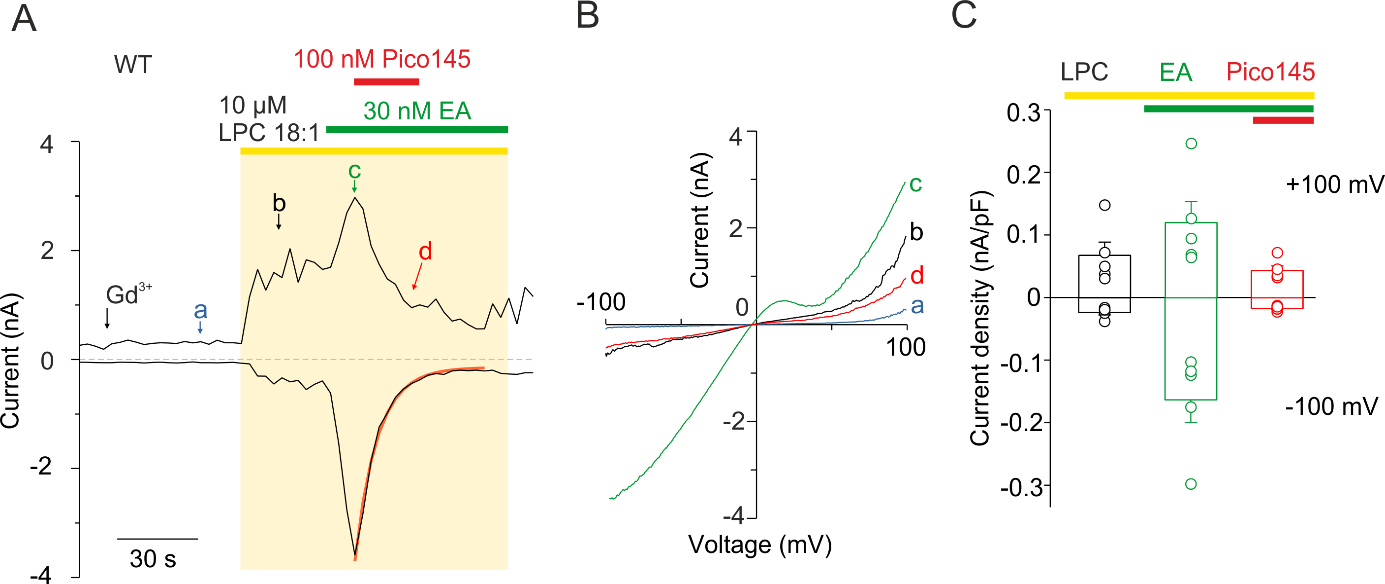


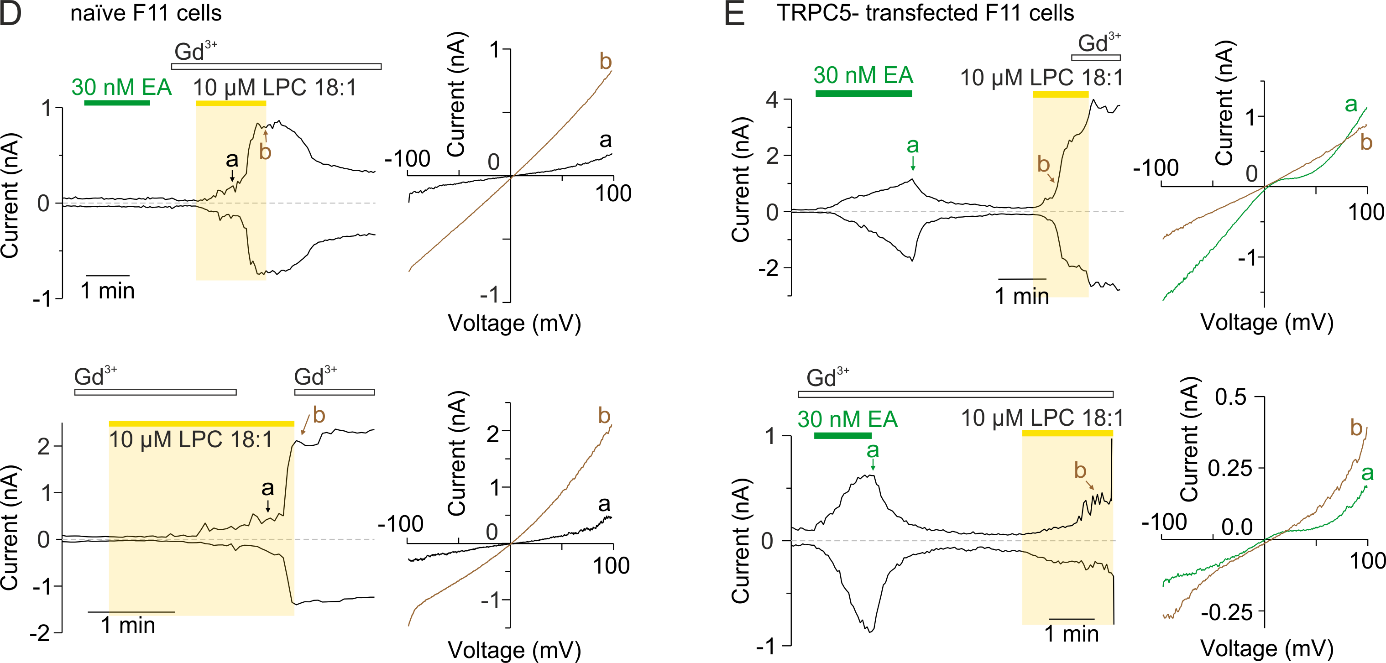


**Supplementary Figure S3** **| Pico145 inhibits** **TRPC5-mediated currents induced by (-)-englerin applied together with LPC 18:1. LPC activates non-TRPC5 mediated currents in F11 cells**

(**A**) Representative whole-cell current measured in the presence of 10 µM LPC 18:1, 30 nM (-)-englerin A (EA) and 100 nM Pico145 from a TRPC5-expressing HEK293T cell in extracellular solution containing 10 µM Gd^3+^ (indicated by vertical arrow). The applications of the compounds are indicated by horizontal bars above the record. A ramp pulse from -100 mV to +100 mV from a holding potential of 0 mV was periodically applied every 3 seconds for 500 ms (protocol shown in Fig. 1A). Amplitudes were measured at -100 mV and +100 mV and plotted as a function of time. (**B**) The current-voltage relations at the time points indicated by the letters in panel A. (**C**) Raw current densities recorded from 5 independent measurements such as shown in panel A. (**D**) Typical whole-cell recordings from naïve F11 cells showing the effect of 10 µM LPC 18:1 in the presence and absence of Gd^3+^. Corresponding current-voltage relationships are shown on the right. (**E**) Representative whole-cell currents induced by (-)-englerin A (EA) measured from TRPC5-transfected F11 cells. Please compare the activation kinetics with EA-induced response in a HEK293T cell shown in Supplementary Fig. S1H. The current-voltage relations at the time points indicated by the letters are shown on the right.

**
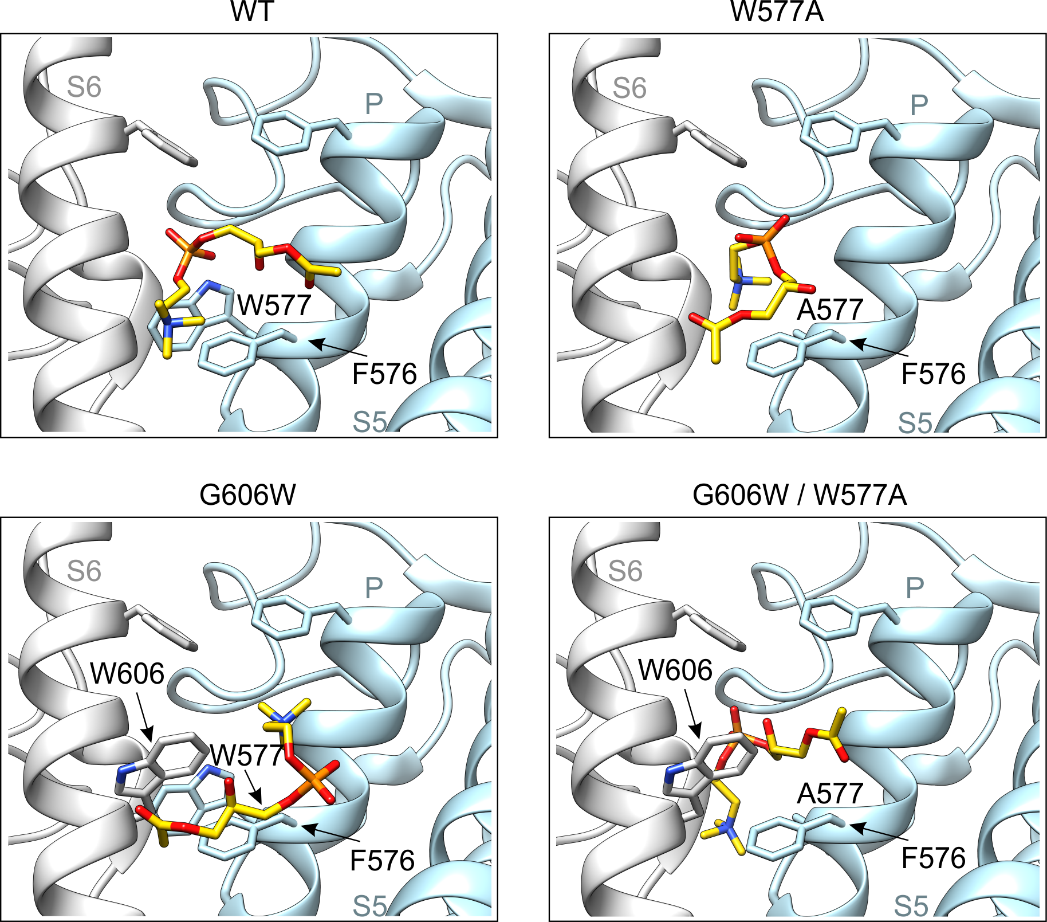
**

**Supplementary Figure S4 | Molecular docking suggesting a possible interaction between the L2 lipid recognition (xanthine-binding) site and LPC.**

Close-up view of the LPC-interacting residues at positions 577, 576 and 606 located between chain A (light gray) and B (light blue) of the TRPC5 structure (PDB ID: 7E4T; the ligand YZY was extracted from the initial structure) and its mutants. The AutoDock Vina tool was used to predict the possible binding modes of LPC to the xanthine binding site of TRPC5 (7E4T) and its mutants. LPC was set as flexible for the docking. In the W577A structure, the LPC head may delve deeper and compensate for the replacement of the robust tryptophan side chain with a small alanine at position 577 (AutoDock docking score -5.2 kcal mol^-1^ versus -5.3 kcal mol^-1^ for wild-type structure). In the G606W mutant, the LPC interaction might be weaker (AutoDock docking score -3.5 kcal mol^-1^) because the binding site is filled by the side chain of the mutated amino acid.


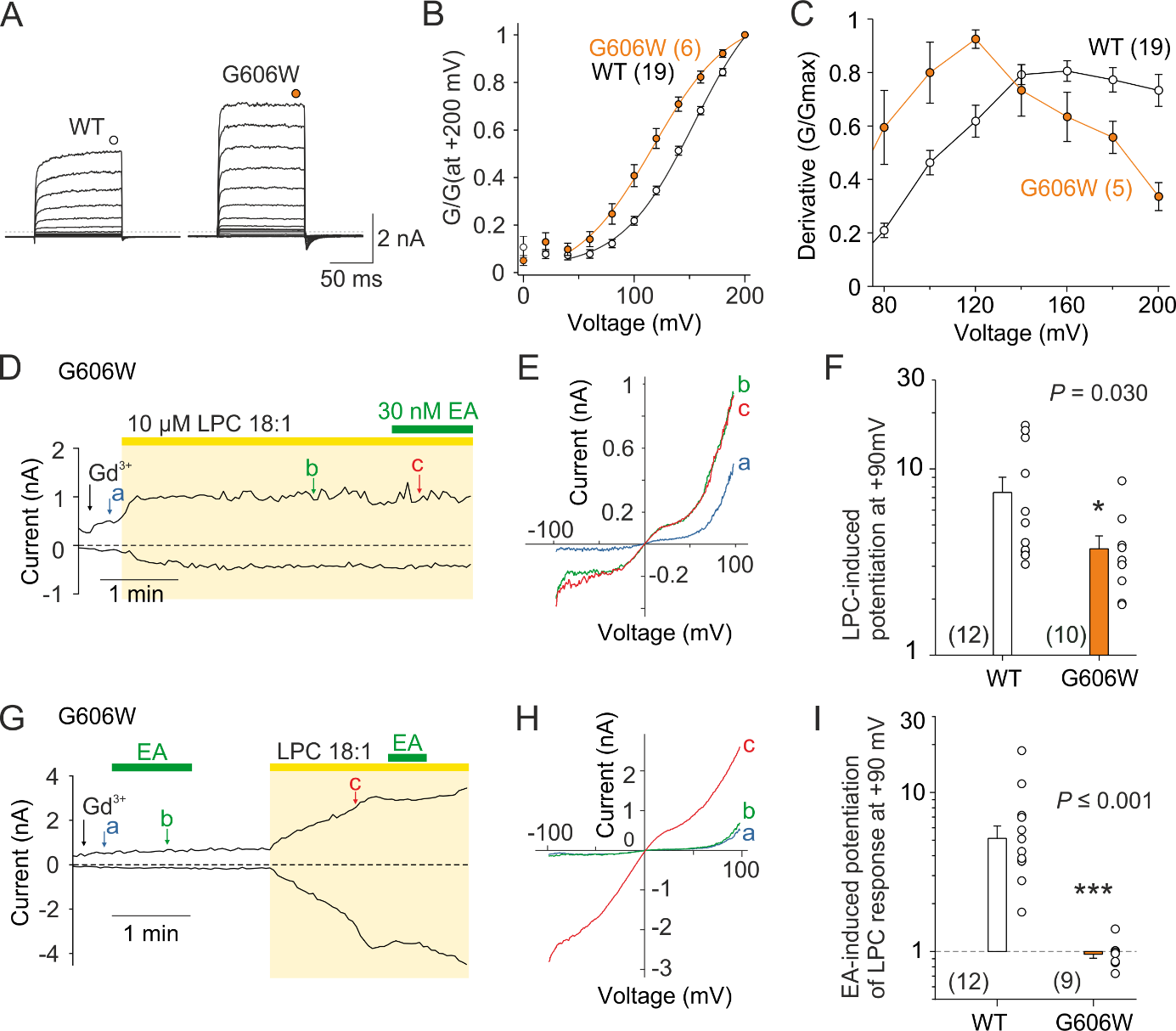


**Supplementary Figure S5** **|** **Mutation G606W produces gain-of-function phenotype and renders TRPC5 insensitive to** (-)-**englerin A**

(**A**) Representative current traces in response to a voltage step protocol (from -80 to +200 mV; 20 mV step) recorded from cells expressing wild-type (WT) or G606W TRPC5 channels as indicated. The currents were recorded after 30-40 second of exposure to extracellular control solution containing 10 μM Gd^3^. (**B**) The average conductance-voltage plots for TRPC5 constructs indicated above, normalized to the maximum response at +200 mV, obtained in extracellular control solution containing Gd^3+^. Steady-state currents were measured at the end of the pulses as indicated by colored symbols atop the records shown in A. The data were fitted by Boltzmann equation over the interval from +40 mV to +200 mV; solid lines). Number of measured cells is indicated in parentheses. Data are mean ± SEM. (**C**) The average normalized derivative of conductances plotted as a function of voltage, measured from TRPC5 and G606W expressing cells in extracellular control solution containing Gd^3+^ as shown in A. The peak of each plot corresponds to the inflection point of the Boltzmann relationship and indicates the value of the half-maximal activation voltage (*V*_50_). Number of biological replicates is indicated in parentheses. The bar graph shows the mean + SEM. (**D**) Representative time course of whole-cell currents induced by 10 μM LPC 18:1 and in the presence of added (-)-englerin A (EA; 30 nM) in the G606W mutant of TRPC5. A ramp pulse protocol as shown in Fig. 1A was applied and amplitudes measured at -100 mV and +100 mV were plotted as a function of time. (**E**) The current-voltage relations at the time points indicated by the letters in panel D. (**F**) The voltage-dependent currents mediated by G606W were potentiated less than those of the wild-type channels. Number of biological replicates is indicated in parentheses. Bar graph shows mean + SEM (*P* = 0.030; Mann-Whitney test). (**G**) Representative time course of G606W-mediated whole-cell currents measured in the presence of 30 nM (-)-englerin A (EA) and then in the presence of 10 μM LPC 18:1. A ramp pulse protocol as shown in Fig. 1A was applied and amplitudes measured at -100 mV and +100 mV were plotted as a function of time. (**H**) The current-voltage relations at the time points indicated by the letters in panel G. (**I**) The effect of 30 nM (-)-englerin A (EA) on LPC-induced currents measured from recordings such as shown in Fig. 2E for wild-type TRPC5 and in D for G606W channels. Number of biological replicates is indicated in parentheses. The bar graph shows the mean + SEM (****P* ≤ 0.001; Mann-Whitney test).


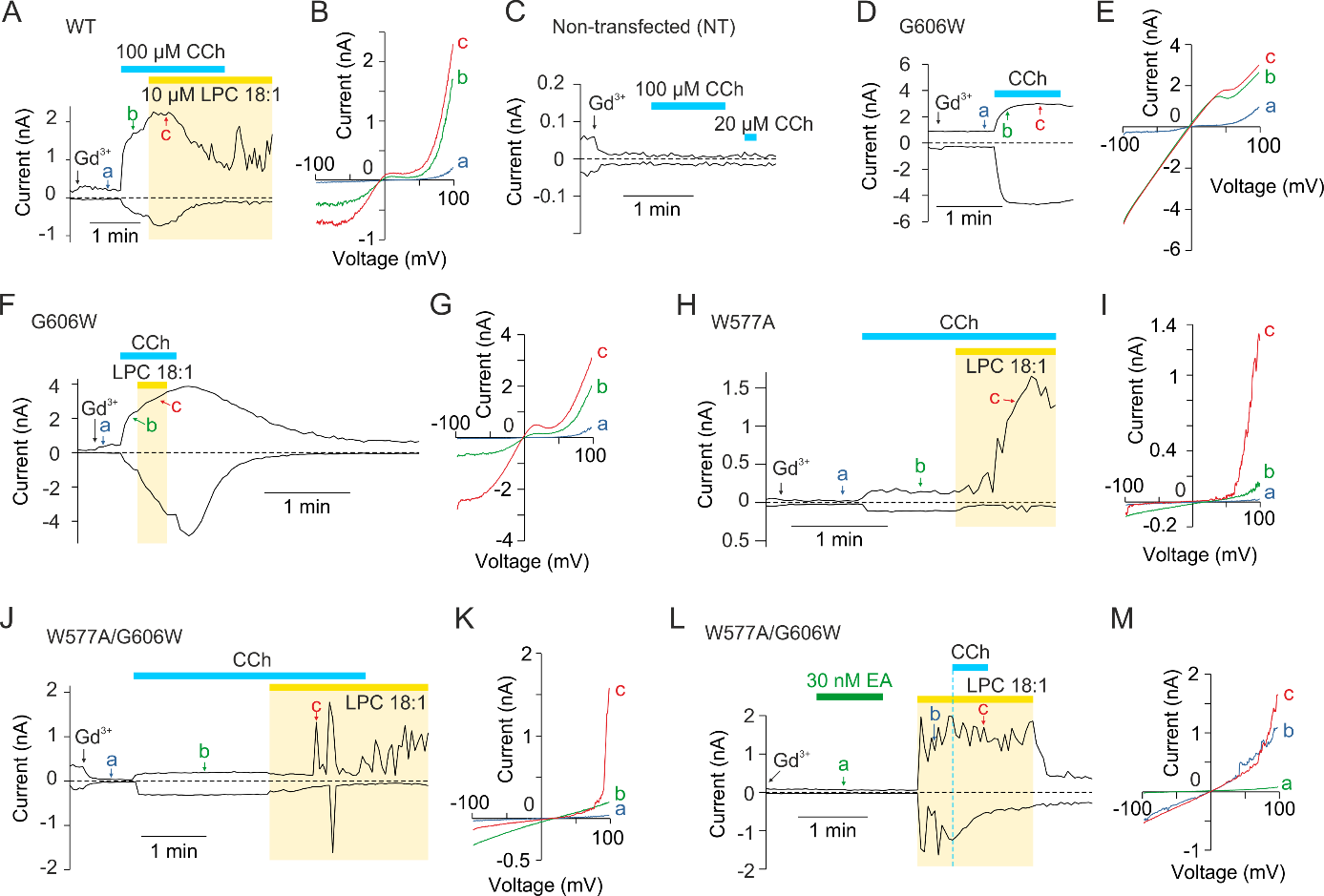


**Supplementary Figure S6** **|** **Effect of carbachol on critical TRPC5 constructs**

(**A**) Representative time course of whole-cell currents induced by 100 µM carbachol (CCh) and 10 µM LPC 18:1 in the wild-type channel in the presence of 10 µM Gd^3+^ (indicated by vertical arrow). A ramp pulse protocol as shown in Fig. 1A was applied and amplitudes measured at -100 mV and +100 mV were plotted as a function of time. (**B**) The current-voltage relations at the time points indicated by the letters in panel A. (**C**) Representative time course of whole-cell currents recorded from non-transfected control HEK293T cell in the presence of 100 µM CCh and 20 µM CCh, measured at -100 mV and +100 mV. (**D**) Representative whole-cell currents recorded from G606W mutant TRPC5 induced by 100 µM CCh. (**E**) The current-voltage relations at the time points indicated by the letters in panel D. (**F**) Representative whole-cell currents recorded from G606W mutant TRPC5 induced by 100 µM CCh and 10 µM LPC 18:1. (**G**) The current-voltage relations at the time points indicated by the letters in panel F. (**H**) Representative time course of whole-cell currents induced by 100 µM CCh and 10 µM LPC 18:1 in the W577A mutant channel. (**I**) The current-voltage relations at the time points indicated by the letters in panel H. (**J**) Representative whole-cell currents recorded from W577A/G606W mutant TRPC5 induced by 100 µM CCh and 10 µM LPC 18:1. (**K**) The current-voltage relations at the time points indicated by the letters in panel J. (**L**) Representative time course of whole-cell currents of the W577A/G606W mutant TRPC5 recorded in the presence of 30 nM (-)-englerin A (EA), 10 µM LPC 18:1 and 100 µM CCh. (**M**) The current-voltage relations at the time points indicated by the letters in panel L.


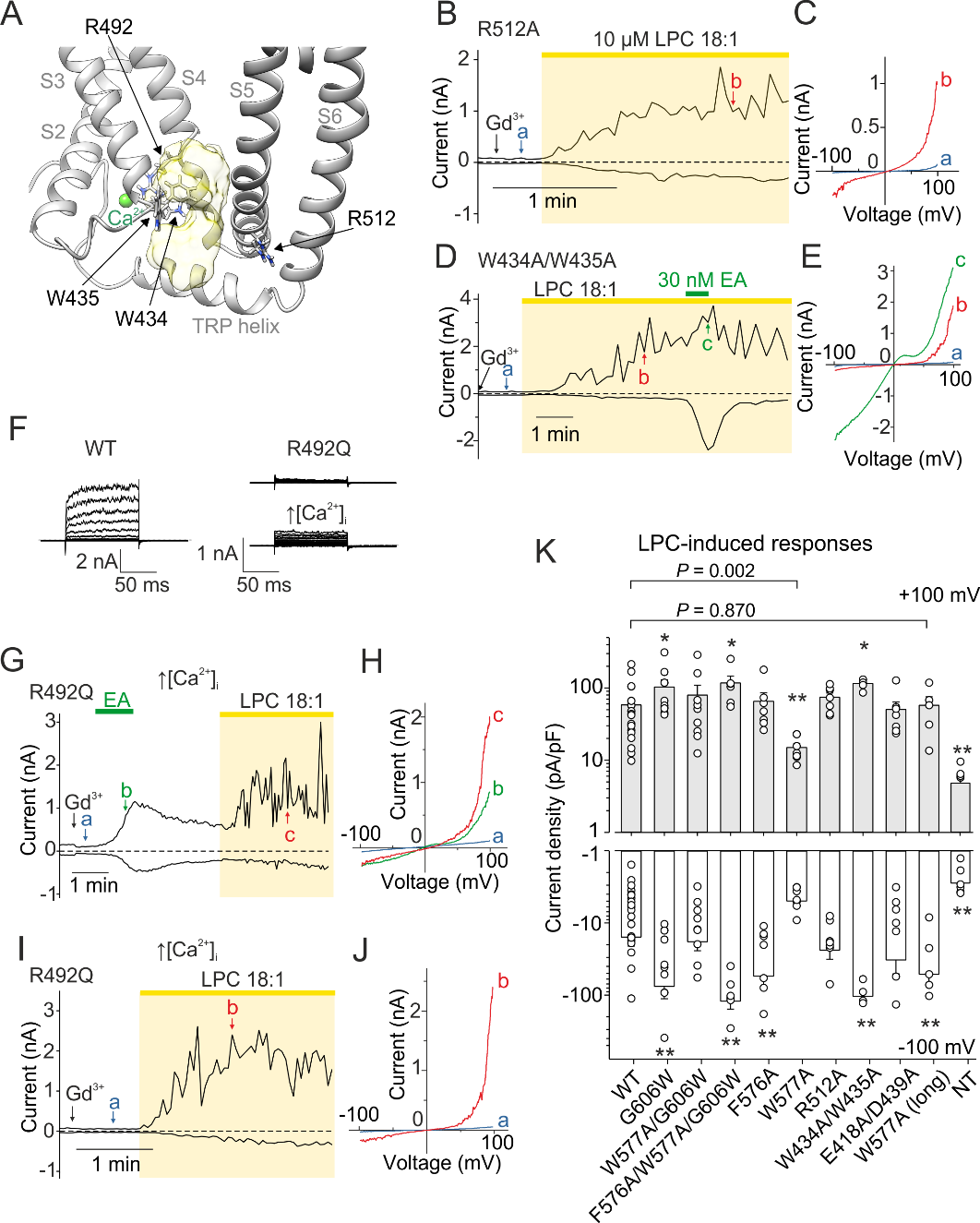


**Supplementary Figure S7 | Mutations of sites potentially involved in voltage-dependent activation of TRPC5 do not eliminate LPC sensitivity**

(**A**) Zoomed-in view of the sensor domain of TRPC5 (PDB ID: 7E4T) with a putative PIP_2_-binding pocket (PIP_2_ indicated as light yellow surface), residues potentially interacting with PIP_2_, W434, W435, and R512. The residue R492 located in the S4 helix is oriented to the interior of the sensor cavity where it contributes to Ca^2+^ coordination. (**B**) Representative time course of whole-cell currents induced by 10 µM LPC 18:1 in the R512A mutant of TRPC5 channel. A ramp pulse protocol as shown in Fig. 1A was applied and amplitudes measured at -100 mV and +100 mV were plotted as a function of time. (**C**) The current-voltage relations at the time points indicated by the letters in panel B. (**D**) Representative time course of LPC-induced currents in the presence of 30 nM (-)-englerin A (EA) in the W434A/W435A mutant of TRPC5. (**E**) The current-voltage relations at the time points indicated by the letters in panel D. (**F**) Representative current traces in response to a voltage step protocol (from -80 to +200 mV; as shown in Fig. 1I) recorded from cells expressing wild-type (WT) or R492Q TRPC5 channels as indicated. The currents were recorded after 30-40 second of exposure to extracellular control solution containing 10 μM Gd^3^. The lower recording of the R492Q mutant was performed with a pipette solution in which concentration of free Ca^2+^ was increased to 100 µM (see Materials and Methods). (**G**, **I**) Representative time course of EA- and LPC-induced whole-cell currents in the R492Q mutant of TRPC5. The recordings were performed with a pipette solution in which concentration of free Ca^2+^ was increased to 100 µM (see Materials and Methods). (**H**, **J**) The current-voltage relations at the time points indicated by the letters in panel G and I, respectively. (**K**) Summarized current densities of whole-cell responses induced by 10 µM LPC 18:1 in the constructs of human TRPC5, measured at +100 mV and -100 mV from recordings such as in panel B, averaged over ~1 min of steady-state LPC application. Number of biological replicates: WT (*n* = 22), G606W (*n* = 10), W577A/G606W (*n* = 9), W577A/F576A/G606W (*n* = 6), F576A (*n* = 7), W577A (*n* = 7), R512A (*n* = 7), W434A/W435A (*n* = 5), E418A/D439A (*n* = 7), non-transfected cells (NT; *n* = 6), and W577A measured with a protocol with prolonged depolarization as shown in Figure 3E (*n* = 5). The bar graphs show mean + SEM (One-Way ANOVA with Dunnett’s multiple comparison test; *, *P* < 0.05; **, *P* < 0.01; versus wild-type TRPC5; unpaired two-tailed *t*-test). At negative membrane potential (-100 mV), currents were significantly larger than currents measured in wild-type channels (WT) in G606W (*P* = 0.001), W577A/F576A/G606W (*P* < 0.001), F576A (*P* = 0.007) and W434A/W435A (*P* < 0.001), and in W577A stimulated by a voltage protocol with a prolonged depolarization phase (W577A-long; *P* = 0.009).


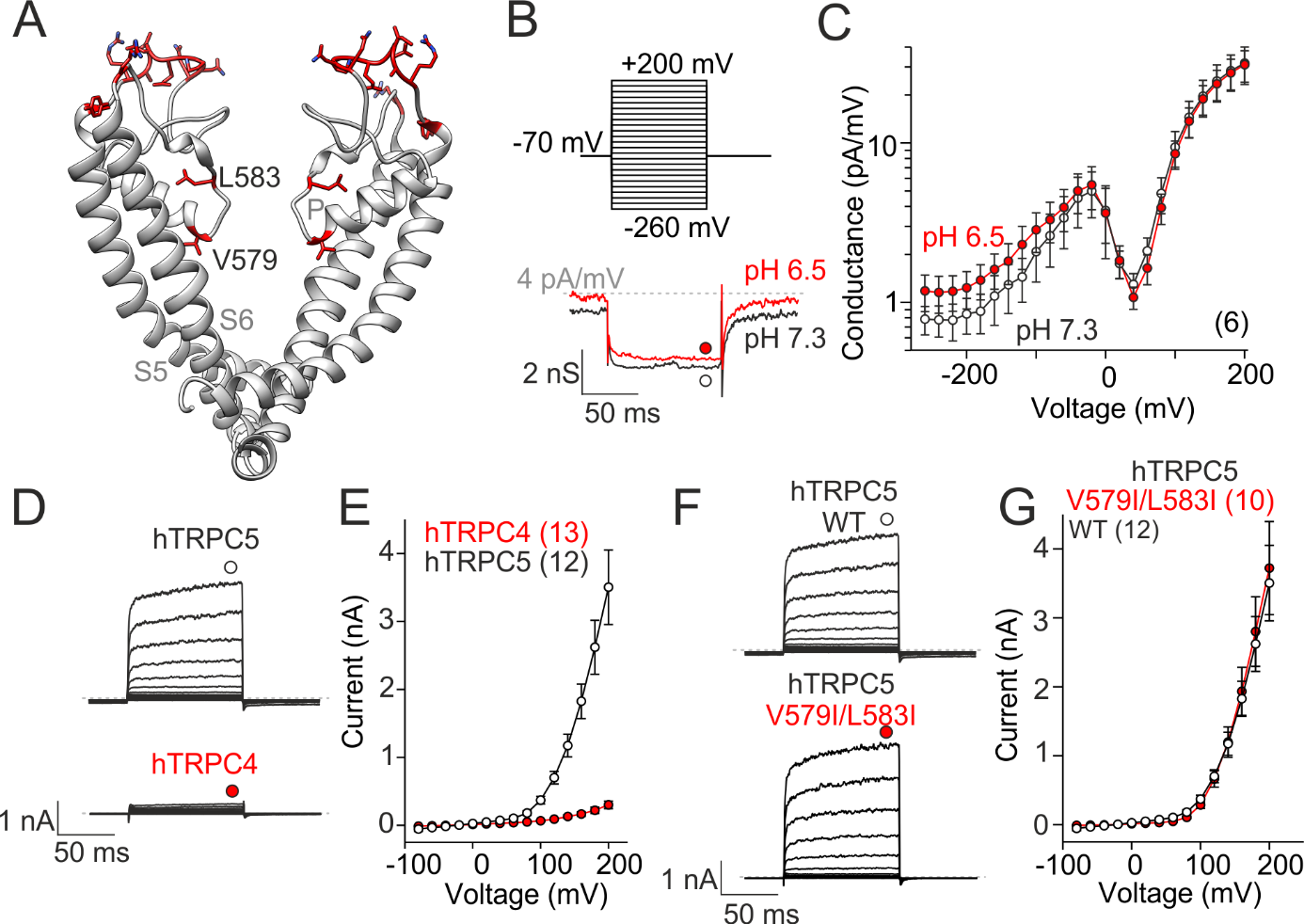


**Supplementary Figure S8 | Voltage does not act through the activation pathway for protons**

(**A**) Side view of the structure of the pore domain of two opposite subunits of human TRPC5 (PDB ID: 7E4T) with the residues that distinguish TRPC5 from TRPC4 highlighted in red. (**B**) Voltage step protocol (20 mV step from -260 to +200 mV) and average conductances measured from current responses at -260 mV from TRPC5-expressing cells. The currents were recorded in control solution containing 10 μM Gd^3+^ at pH 7.3 (black trace) or 6.5 (red trace). Steady-state currents were measured at the end of the pulses as indicated by colored symbols. (**C**) The average conductance-voltage plots obtained using voltage step protocol in panel B. Note that the relationship exhibits a steady state level at deeply hyperpolarized potentials (< ‑200 mV) under both conditions. Number of measured cells is indicated in parentheses. Data are mean ± SEM. (**D**) Average current traces in response to a voltage step protocol (as shown in Fig. 1I) recorded from 12 cells expressing TRPC5 and 13 cells expressing TRPC4 channels as indicated. (**E**) The average current-voltage plots. Steady-state currents were measured at the end of the pulses as indicated by colored circles shown in panel D. Number of measured cells is indicated in parentheses. Data are mean ± SEM. (**F**) Average current traces in response to a voltage step protocol (as shown in Fig. 1I) recorded from 12 cells expressing wild type (WT) or 10 cells expressing V579I/L583I TRPC5 channels. (**G**) The average current-voltage plots. Steady-state currents were measured at the end of the pulses as indicated by colored circles shown in panel F. Number of measured cells is indicated in parentheses. Data are mean ± SEM.


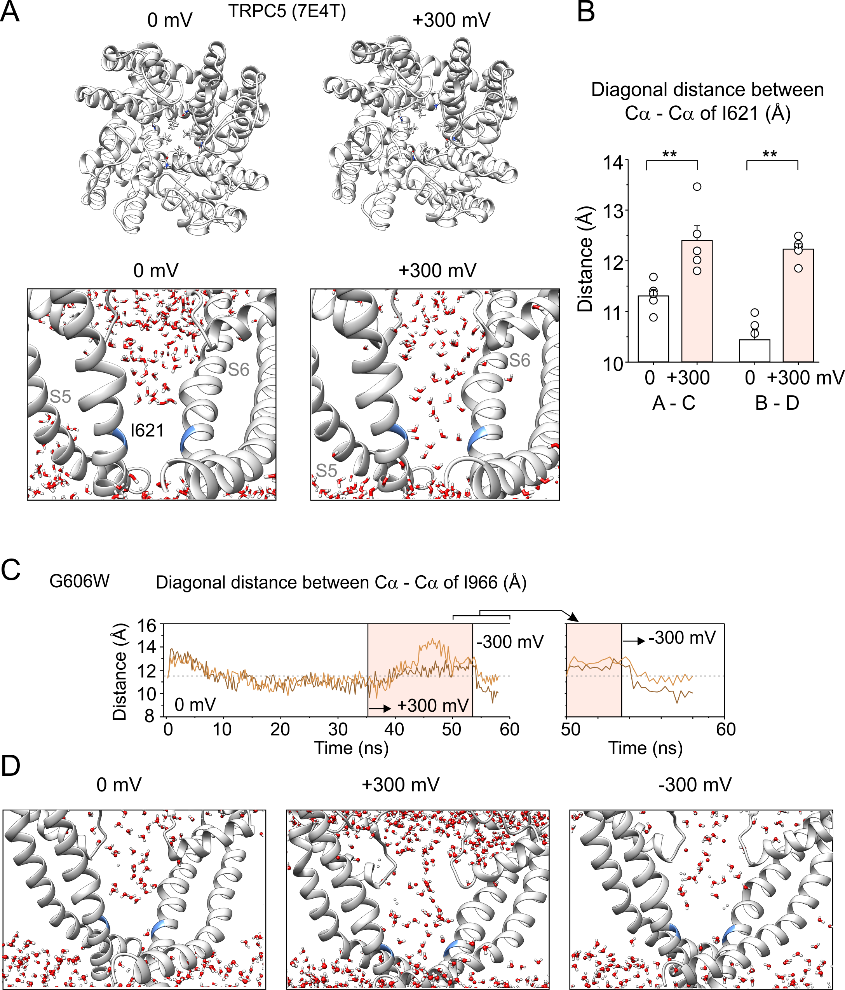


**Supplementary Figure S9 | Molecular dynamics simulations of TRPC5 during depolarization and hyperpolarization**

(**A**) Top view of the pore region of human TRPC5 (PDB ID: 7E4T) with the side chains of residues forming the lower gate I621 and N618 shown as sticks. Snapshots show the structural rearrangement induced by depolarization of membrane potential from 0 mV (left) to +300 mV (right). Below, enlarged side view of the pore domain structure of two opposite subunits B and D of human TRPC5 (PDB ID: 7E4T) with the position of I621 indicated in blue. Subunits A and C are omitted for clarity. Snapshots from the molecular dynamics simulations at 0 mV (left) and at +300 mV (right) indicate that water molecules can penetrate and reside in the lower pore during depolarization. (**B**) Statistics of distances measured diagonally between the backbone Cα of two opposite residues I621 in chains A-C and B-D. The distances were averaged over the period of 20-30 ns for 0 mV and 45-55 ns for +300 mV from the trajectories of 5 independent MD simulations. (**C**) Instantaneous distance measured diagonally between the backbone Cα of I621 in S6 of chains A and C (darker colored line), and chains B and D (lighter colored line), plotted as a function of simulation time for G606W mutant. The colored (light orange) area indicates the time when depolarization at +300 mV was applied. The horizontal dashed line denotes the diagonal distance between the backbone Cα of isoleucines 621 measured from the initial structure (11.51 Å). The time period when hyperpolarization -300 mV was applied is enlarged on the right. (**D**) Enlarged side view of the pore domain structure of two opposite subunits B and D of the G606W mutant of human TRPC5 (PDB ID: 7E4T) with the position of I621 indicated in blue. Subunits A and C are omitted for clarity. Snapshots from the molecular dynamics simulations at 0 mV (left) and at +300 mV (middle) and -300 mV (right) indicate that water molecules can penetrate and reside in the lower pore during depolarization and this process is reversible upon hyperpolarization to -300 mV.

**
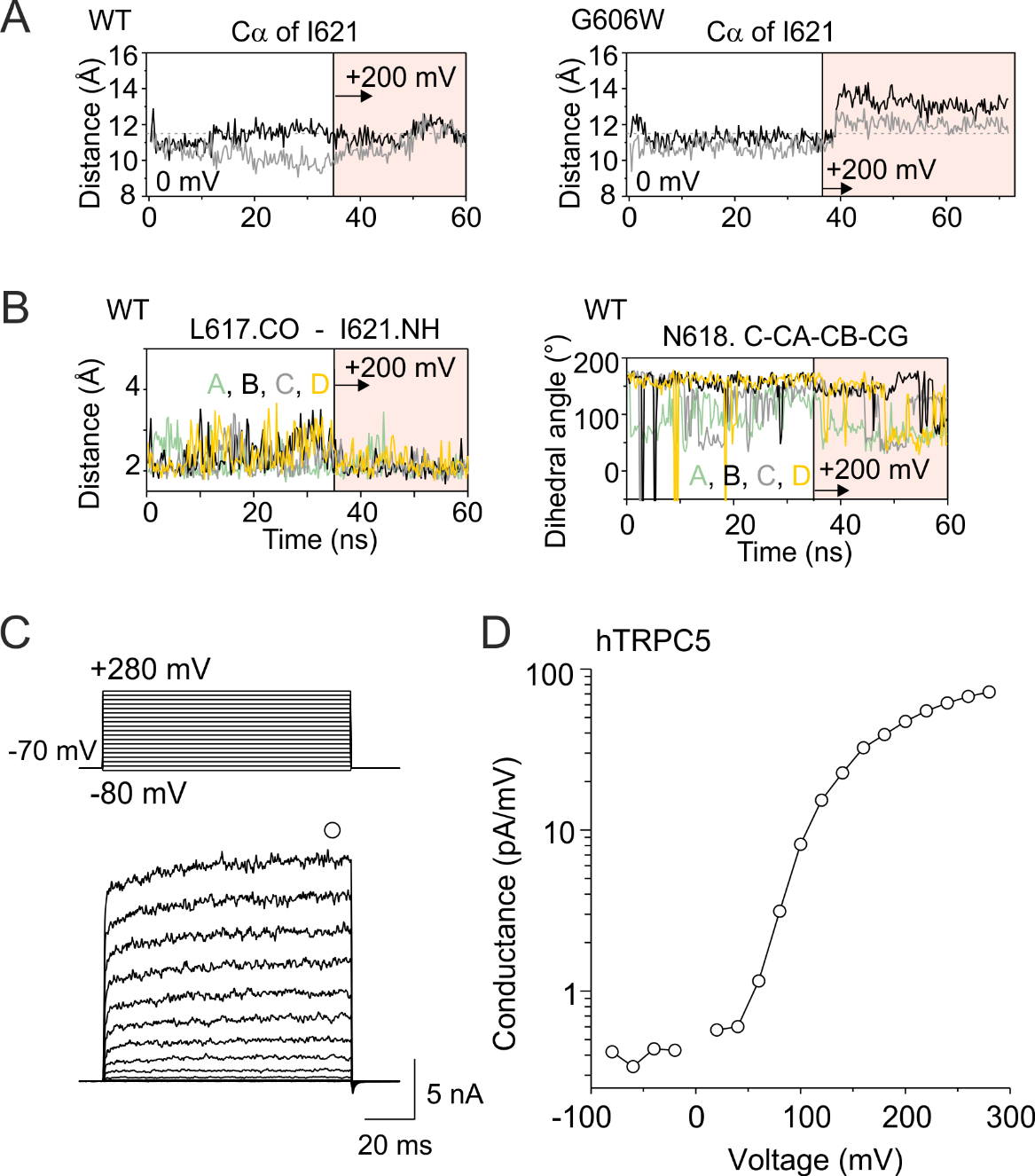
**

**Supplementary Figure S10 | Molecular dynamics simulations at +200 mV**

(**A**) Instantaneous distance measured diagonally between the backbone Cα of I621 in S6 of chains A and C (darker colored line), and chains B and D (lighter colored line), plotted as a function of simulation time for wild-type structure (7E4T; left) and G606W (right) at 0 mV and at +200 mV. The colored (light orange) area indicates the time when depolarization at +200 mV was applied. The horizontal dashed line denotes the diagonal distance between the backbone Cα of isoleucines 621 measured from the initial structure (11.51 Å). (**B**) Left, distance between the backbone CO group of L617 and NH group of I621, plotted as a function of simulation time for the chains A, B, C and D for wild-type structure. The colored area indicates the time when depolarization at +200 mV was applied. Right, changes in the dihedral angle values for the side-chain of N618 (C-CA-CB-CG), plotted as a function of simulation time for the chains A, B, C and D for wild-type structure. (**C**) Voltage-induced TRPC5 currents recorded in extracellular control solution using the voltage step protocol from -80 mV to +280 mV (20 mV steps) indicated above the record. (**D**) The conductance-voltage plot obtained from the recording shown in panel C.


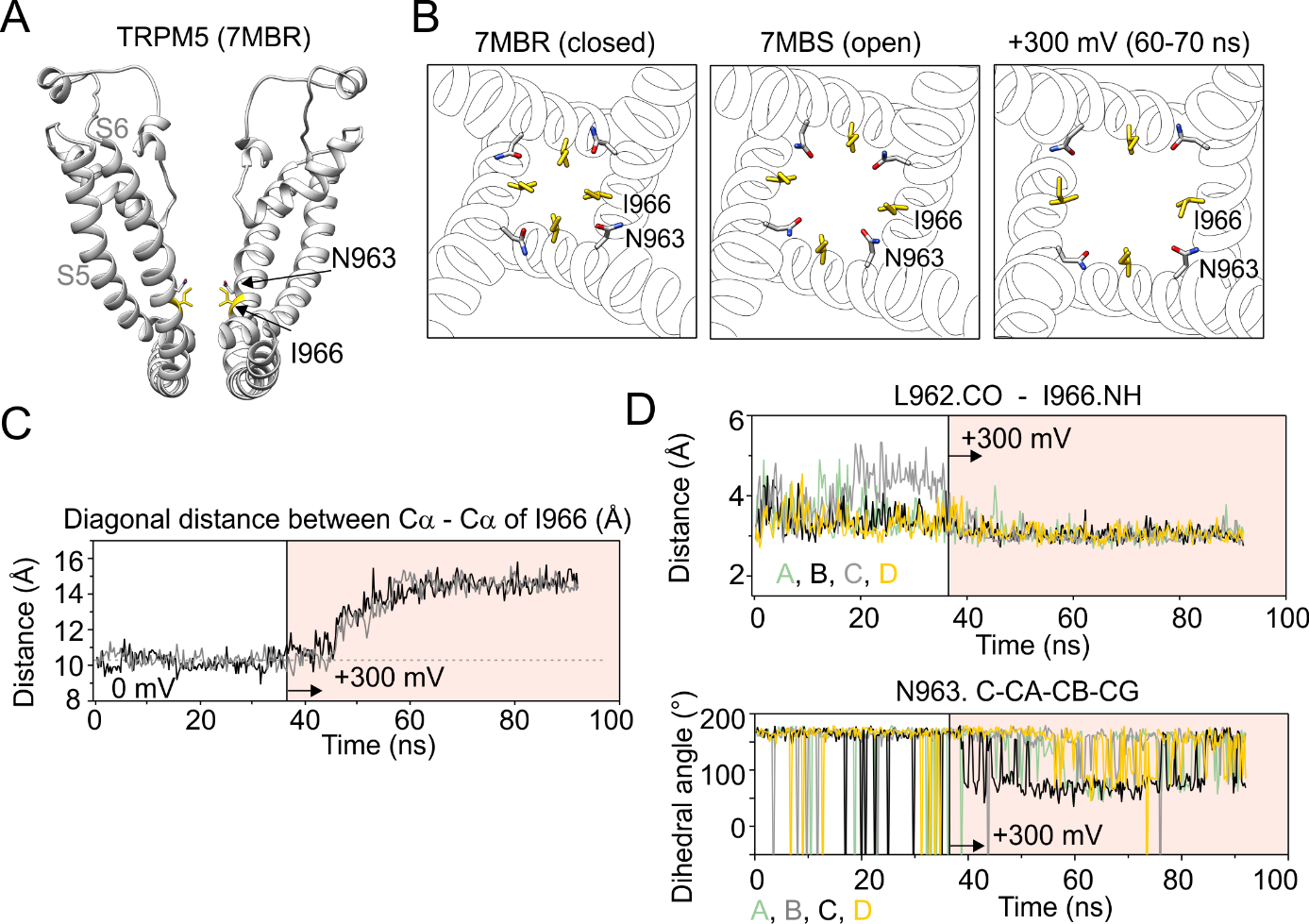


**Supplementary Figure S11 | Molecular dynamics simulations of TRPM5 during depolarization**

(**A**) The simulation approach was validated using the closed state structure of TRPM5 channel. Side view of the pore region of zebrafish TRPM5 (PDB ID: 7MBR) with the side chains of residues forming the lower gate I966 and N963 shown as sticks. (**B**) Top view of the pore region of TRPM5 in a closed (PDB ID: 7MBR) and open (7MBS) conformation. The averaged structure (over the period of 70-80 ns) obtained from the molecular dynamics simulations at +300 mV (right) is shown for comparison. (**C**) Instantaneous distance measured diagonally between the backbone Cα of two opposite residues I966 that form the lower hydrophobic gate in TRPM5 (corresponding to I621 in TRPC5). (**D**) The distances between the backbone CO group of L962 and NH group of I966, and the side-chain orientation of N963 (corresponding to N618 in TRPC5) exhibited qualitatively analogous changes upon depolarization to +300 mV as in the MD simulation with TRPC5 (please compare with Fig. 5C, E and G).

**Supplementary Movie 1** **| Molecular dynamics simulations of TRPC5 during depolarization**

Conformational changes and partial opening of the TRPC5 channel induced by depolarization of membrane potential from 0 mV to +300 mV.
